# A strong preference for the TA/TA dinucleotide step discovered for an acridine-based, potent antitumor dsDNA intercalator, C-1305: NMR-driven structural and sequence-specificity studies

**DOI:** 10.1038/s41598-020-68609-8

**Published:** 2020-07-16

**Authors:** Tomasz Laskowski, Witold Andrałojć, Jakub Grynda, Paulina Gwarda, Jan Mazerski, Zofia Gdaniec

**Affiliations:** 1 Department of Pharmaceutical Technology and Biochemistry, Faculty of Chemistry, Gdańsk University of Technology, Gabriela Narutowicza Str. 11/12, 80-233 Gdańsk, Poland; 2 Institute of Bioorganic Chemistry, Polish Academy of Sciences, Zygmunta Noskowskiego Str. 12/14, 61-704 Poznań, Poland

**Keywords:** DNA, Solution-state NMR, Drug discovery and development, Molecular dynamics, Drug development

## Abstract

Triazoloacridinone C-1305, a potent antitumor agent recommended for Phase I clinical trials, exhibits high activity towards a wide range of experimental colon carcinomas, in many cases associated with complete tumor regression. C-1305 is a well-established dsDNA intercalator, yet no information on its mode of binding into DNA is available to date. Herein, we present the NMR-driven and MD-refined reconstruction of the 3D structures of the d(CGATATCG)_2_:C-1305 and d(CCCTAGGG)_2_:C-1305 non-covalent adducts. In both cases, the ligand intercalates at the TA/TA site, forming well-defined dsDNA:drug 1:1 mol/mol complexes. Orientation of the ligand within the binding site was unambiguously established by the DNA/ligand proton-proton NOE contacts. A subsequent, NMR-driven study of the sequence-specificity of C-1305 using a series of DNA duplexes, allowed us to confirm a strong preference towards TA/TA dinucleotide steps, followed by the TG/CA steps. Interestingly, no interaction at all was observed with duplexes containing exclusively the AT/AT, GG/CC and GA/TC steps.

## Introduction

**Figure 1:**
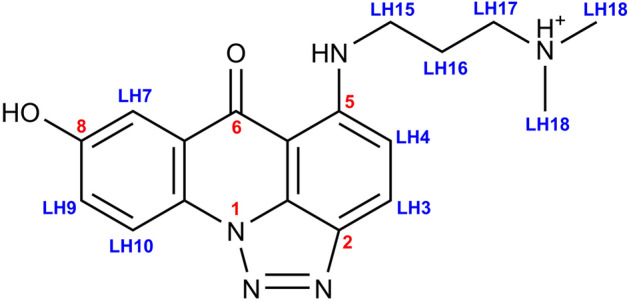
The structure of 5-[[3-(dimethylamino)propyl]amino]-8-hydroxy-6H-v-triazolo[4,5,1-de]acridin-6-one, codenamed C-1305, with a protonated terminal tertiary nitrogen atom.

DNA-directed, rational antineoplastic agent development has made an extensive use of the acridine pharmacophore^1^. Among many diverse families of acridine derivatives, development of a series of novel triazoloacridinones with potent antitumor activities has been started some time ago^2^. The most active triazoloacridinone derivative obtained to date, 5-[[3-(dimethylamino)propyl]amino]-8-hydroxy-6H-v-triazolo[4,5,1-de]acridin-6-one, codenamed C-1305 (Fig. 1), showed high antitumor activity towards a wide range of different experimental tumors in vitro and in vivo, including both murine and human colon carcinomas, which in most cases was associated with complete tumor regression^3^. Induction of apoptosis in human leukemia cells after uptake of C-1305 has also been demonstrated^4,5^. Its interaction with several molecular targets^6,7^, as well as its metabolism^8–10^, were extensively studied. For instance, this compound was shown to be a topoisomerase II poison, which stabilizes unusually toxic covalent complexes between DNA and the enzyme^11^. It was also demonstrated that C-1305 is a viable double stranded DNA (dsDNA) intercalator, yet no sequence-specificity of this potential drug was revealed since UV, CD and ELD studies have shown that it intercalated into ctDNA, p(dAdT)_2_ and p(dGdC)_2_ polymers at the same ratio^12^. Moreover, chemical probing with DEPC, combined with molecular modelling studies suggested that C-1305 is able to induce substantial distortions of a dsDNA duplex while intercalating into the GGG triplets, which is a unique effect among the known topoisomerase II inhibitors. Yet, this result was strongly dependent on the protonation of the drug and was observed only in pH > 7.5, in which C-1305 is believed to exist mostly in a zwitterionic form with deprotonated 8-OH hydroxyl group and protonated tertiary nitrogen atom at the end of the sidechain^12^ (Fig. 1). Nevertheless, no structural studies on the mode of intercalation of C-1305 into DNA were performed as it was merely assumed that the binding occurs from the minor groove of the helix. That statement was made on the basis of the structural resemblance of C-1305 to other known minor-groove intercalators^12^. It might be very misleading since there are several known examples of other, structurally close acridine-based dsDNA intercalators, such as 9-amino-DACA and its derivatives^13,14^, which bind from the major groove of the helix.

Rational design of new drugs requires the knowledge of their molecular mechanism of action. We believe that the complete understanding of the structural aspects of the dsDNA:C-1305 non-covalent adducts formation is crucial for further, biochemically-oriented studies and for the synthesis of new compounds, potentially exhibiting better pharmacological properties. Our previous studies on a C-1305 analogue—imidazoacridinone C-1311^15,16^—have suggested that the apparent lack of sequence-specificity of this drug family might not be the case. Thus, we decided to revisit also this aspect using NMR spectroscopy and molecular dynamics (MD) modelling.

Herein, we report the three-dimensional structures of the d(CGATATCG)_2_:C-1305 and d(CCCTAGGG)_2_:C-1305 1:1 mol/mol complexes, fully and unambiguously reconstructed on the basis of 2D NMR experiments and refined by molecular dynamics simulations restrained by NOESY-derived data. Our structures demonstrate that, for these DNA sequences, C-1305 intercalates exclusively at the central 5′-TA-3′/5′-TA-3′ dinucleotide step (from now on denoted in a shorthand notation TA/TA). Furthermore, we also present a detailed, NMR-driven study on the sequence-specificity of C-1305, providing a completely new insight into the drug’s mode of binding into dsDNA.

## Results

### Oligonucleotide sequence selection

**Figure 2:**
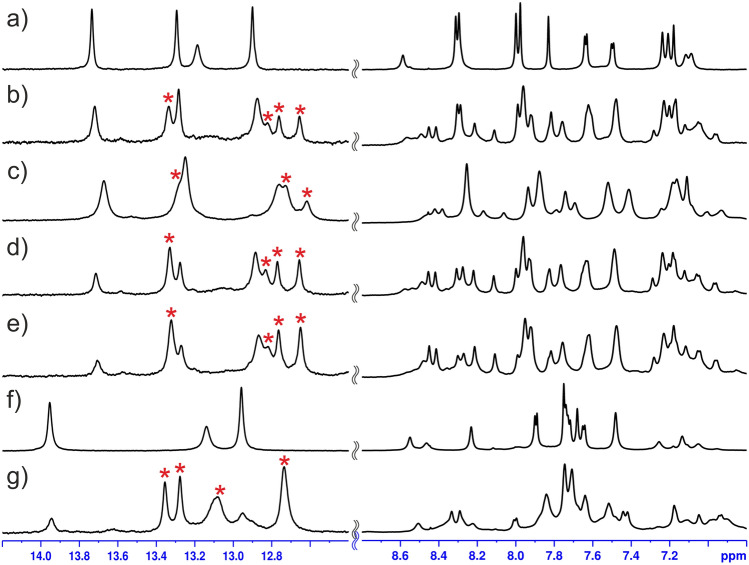
The optimization of the experimental conditions for the formation of the d(CGATATCG)_2_:C-1305 (**D2L**) and d(CCCTAGGG)_2_:C-1305 (**D3L**) complexes: (a) uncomplexed **D2** duplex, (b) **D2L**,1:1 mol/mol in conditions (I), c_DNA_ = 125 µM, (c) **D2L**, 1:1 mol/mol in conditions (I), c_DNA_ = 1.25 mM, (d) **D2L**, 1:1 mol/mol in conditions (II), c_DNA_ = 1.25 mM, (e) **D2L**, 1:1.25 mol/mol in conditions (II), c_DNA_ = 1.25 mM, (f) uncomplexed **D3** duplex, (g) **D3L**, 1:1.5 mol/mol in conditions (II), c_DNA_ = 1.25 mM. The imino proton resonances raised by the formation of the complexes are labelled with asterisk (*).

The investigation started with screening for an oligonucleotide sequence able to form a well-defined complex with C-1305. In the light of some previous results^12^, the d(CCCGGG)_2_ duplex, from now on referred to as **D1**, was chosen assuming that upon addition of C-1305 it would produce a stable, strictly defined non-covalent complex. Such an adduct would possibly enable a spectroscopic confirmation of the previously reported, unique changes in DNA topology, caused by C-1305 intercalating inside a guanosine triplet^12^. Unfortunately, the resulting d(CCCGGG)_2_:C-1305 complex turned out to be too weakly interacting and too labile to produce a set of good quality NMR spectra indispensable for structure determination studies (Supplementary Figure S1). On the second approach, basing on some previous findings regarding sequence specificity of a closely related pro-drug C-1311^16^, the study was focused on a GA/TC-containing palindromic octamer, expecting that it would be able to produce a symmetrical, DNA:drug 1:2 mol/mol complex. The choice fell on the d(CGATATCG)_2_ duplex, from now on referred to as **D2**, which is an extended version of the d(CGATCG)_2_ and d(CGTACG)_2_ duplexes—the standard palindromes widely used in the structural studies on the DNA:drug intercalation complexes^13,17–19^. The d(CGATATCG)_2_ duplex (Fig. 2a) indeed formed a very well-defined complex with triazoloacridinone C-1305 (Fig. 2b), yet—as 2D NMR data has later shown—with 1:1 mol/mol stoichiometry. Furtherly, basing on the sequence-specificity studies described further in the text, it was discovered that insertion of the 5′-TA-3′ dinucleotide step at the very centre of the d(CCCGGG)_2_ duplex radically enhanced its ability to bind C-1305. The resulting d(CCCTAGGG)_2_ duplex, later on referred to as **D3** (Fig. 2f), in the presence of C-1305 produced particularly well-defined non-covalent adduct, which also—proven by the 2D NMR data—displayed 1:1 mol/mol stoichiometry (Fig. 2g).

### Reference NMR data

Standard 2D NMR studies were conducted on the **D2** and **D3** octamers, consisting of NOESY, TOCSY and ^1^H-^13^C-HSQC experiments in conditions with a close-to-physiological pH value (I), namely: 10 mM phosphate buffer, pH = 7.5, 150 mM NaCl (H_2_O and D_2_O solutions). Assignment of the ^1^H and ^13^C resonances was carried out using standard procedures^20–22^. Reference ^1^H NMR data for triazoloacridinone C-1305 (NOESY, TOCSY) were also acquired in the aforementioned experimental conditions.

### Optimization of the experimental conditions

Experimental conditions were optimized for the **D2** octamer and then applied to the rest of the examined duplexes.

Low concentrations of the **D2** duplex (~ 125 µM) were initially investigated in conditions (I). In such setup, d(CGATATCG)_2_ oligomer and 1 molar equivalent of C-1305 formed a non-covalent complex, exhibiting only one spectral form in the solution (Fig. 2b). High specificity of ligand binding was deduced upon the appearance of new, narrow ^1^H resonances corresponding to a single form of the complex. Signal integration revealed that around 50% of total DNA amount was complexed by C-1305. However, the total DNA concentration was too low to produce viable 2D NMR spectra. Surprisingly, increasing the concentration while maintaining DNA/ligand proportions (1.25 mM concentration of the DNA material, 1 molar equivalent of C-1305) resulted in a significant broadening of the proton resonances (Fig. 2c). Even more unexpectedly, higher concentrations also slightly shifted the system’s equilibrium towards the complex dissociation.

A detailed investigation (see Supplementary Data for details) allowed to conclude that this behavior is related to the pronounced self-association of the ligand. Aggregated ligand molecules are unsuitable for intercalation, yet instead they interact non-specifically with DNA, leading to line broadening. This problem could ultimately be alleviated by alteration of the experimental conditions (see Supplementary Data). The optimal experimental conditions for the d(CGATATCG)_2_:C-1305 complex formation were identified as (II): 2.5 mM cacodylate buffer, pH = 5.0, 10 mM NaCl. In conditions (II), an ^1^H NMR spectrum was obtained for the sample of 1.25 mM concentration of the DNA plus 1 molar equivalent of the ligand (Fig. 2d) in which the protons of the complex displayed almost the same chemical shifts as in the spectrum recorded for the sample in conditions (I) (Fig. 2b). Even the imino proton resonances, potentially vulnerable to the changes of the pH of the solution, have not altered their positions. Therefore, it was concluded that the structures of the both d(CGATATCG)_2_:C-1305 complexes, obtained in different (I and II) experimental conditions, were identical. Moreover, it was possible to add an extra 0.25 molar equivalent of the ligand, which shifted the equilibrium of the system towards the complex formation, without broadening the DNA resonances. In such a setup, the well-defined d(CGATATCG)_2_:C-1305 complex, from now on referred to as **D2L**, became a dominant spectral form in the solution (~ 70% of total DNA amount, Fig. 2e).

Interestingly, in the same setup, the d(CCCTAGGG)_2_:C-1305 complex, further on referred to as **D3L** (Fig. 2g), displayed higher fraction of DNA bound by ligand in comparison to **D2L**, unveiling that **D3** duplex exhibits higher affinity towards C-1305 than the **D2** duplex.

In the end, optimization of the experimental conditions, enabling a significant upscale in total DNA and ligand concentrations, allowed to obtain good quality 2D NMR spectra for the **D2L** and **D3L** complexes.

### 2D NMR studies of the d(CGATATCG)2:C-1305 (D2L) complex

**Figure 3:**
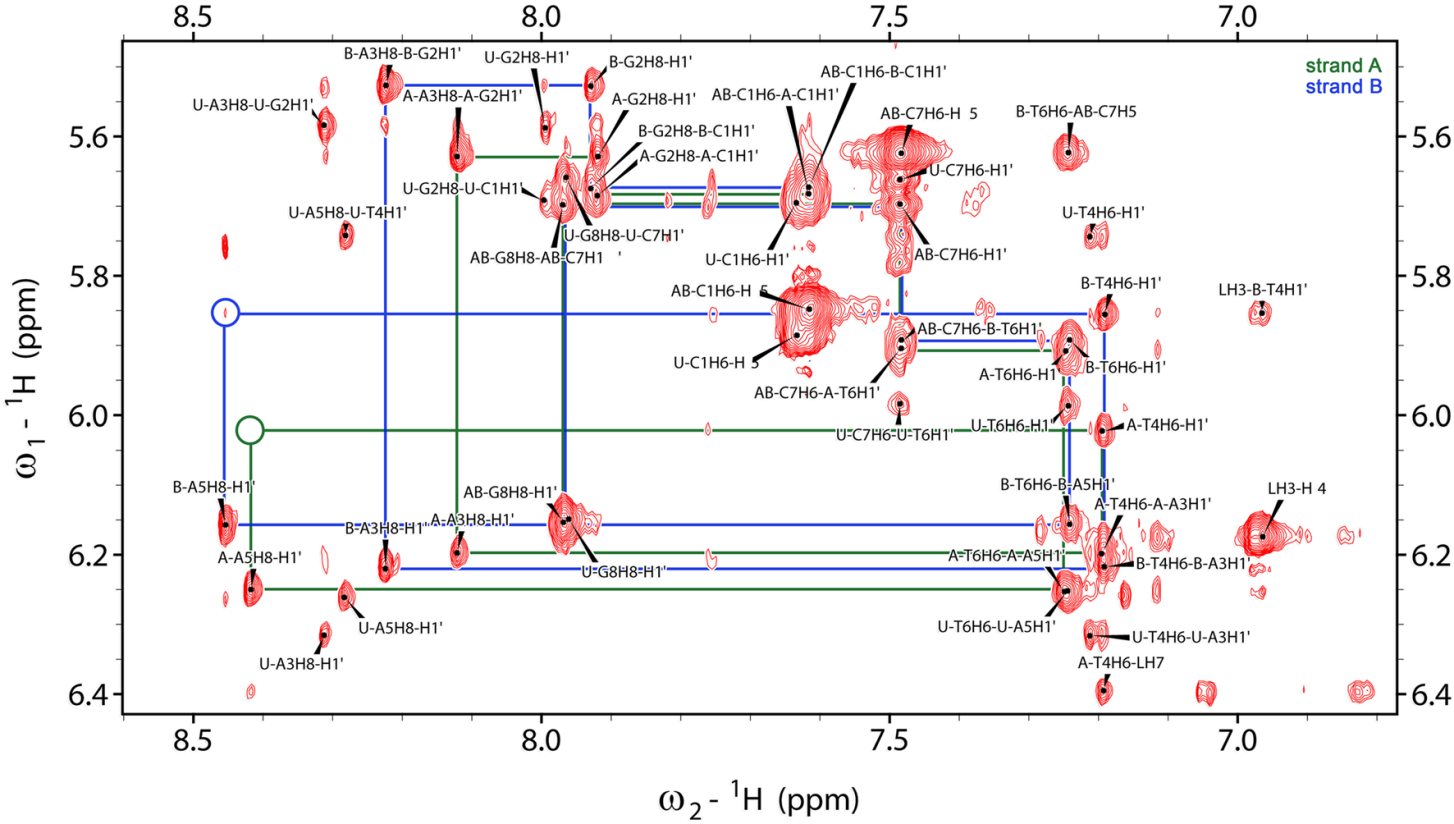
H6/H8-H1′ NOESY-walks for the d(CGATATCG)_2_:C-1305 complex, depicted as green (strand A) and blue (strand B) lines. Missing/extremely weak correlations are marked in circles. This figure was prepared with NMRFAM-SPARKY 1.3^38^.

**Figure 4:**
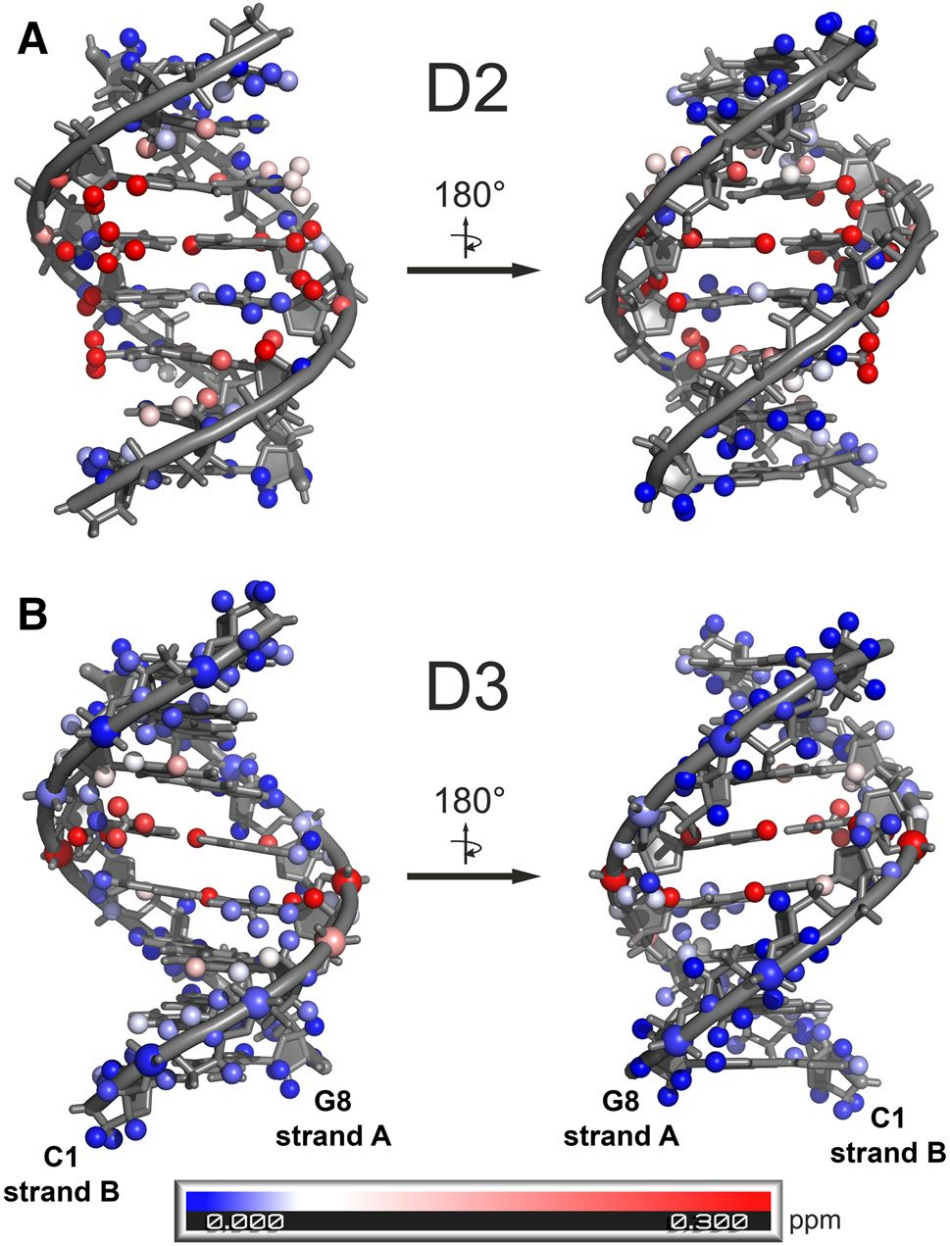
Absolute values of the chemical shift changes (Δδ, ppm) of the DNA resonances upon (**A**) d(CGATATCG)_2_:C-1305 (**D2L**) and (**B**) d(CCCTAGGG)2:C-1305 (**D3L**) complex formation. For a full list of δ and Δδ, please consult Supplementary Tables S1 and S2. PyMOL, version 1.8^39^ was used for visualization.

Assignment of the ^1^H resonances to the d(CGATATCG)_2_:C-1305 complex was performed in a similar fashion as it was done for the non-complexed dsDNA and the free ligand. However, the NMR spectra were severely more complicated, since two distinctive forms of both DNA and C-1305 were present in solution in slow chemical exchange. Moreover, the intercalation of the ligand into DNA duplex broke the symmetry of the helix, which manifested in doubling of ^1^H resonances of most of the residues of the complex. As a result, two DNA strands of the complex needed to be traced separately and were named **D2L-A** and **D2L-B**, respectively (Fig. 3). Nevertheless, despite severe spectral crowding, it was possible to assign all the aromatic base protons of the complex, as well as most of the deoxyribose resonances, namely H1′, H2′, H2″ and H3′ (please consult Supplementary Table S1 for more detailed information). The H4′ and H5′/H5″ resonances were also partially assigned. Due to severe signal overlap in 2D NMR spectra, performing a complete assignment of these protons was not possible. Crucially, all proton resonances of the bound ligand were also unambiguously identified. The chemical shift changes of the DNA resonances upon complex formation are presented in Fig. 4a.

The above described analysis enabled finding over a twenty DNA/ligand intermolecular NOEs, which are listed in Table 1. Most of these NOEs were the contacts between the aromatic protons of the ligand and the protons of the T4 and A5 residues of the both DNA strands. This finding, along with the broken symmetry of the dsDNA octamer and the observed 1:1 dsDNA:ligand molar ratio, clearly suggested that the ligand should be located at the centre of the DNA duplex. The resulting conclusion was well supported by the fact that in the H6/H8-H1′ NOESY-walks (Fig. 3), traced for the both DNA strands of the complex, the correlations T4H1′/A5H8 were either not observed (chain **D2L-A**) or were extremely weak (chain **D2L-B**).

**Table 1:** Observed d(CGATATCG)_2_:C-1305 (**D2L**) and d(CCCTAGGG)_2_:C-1305 (**D3L**) intermolecular NOE contacts. The intensities were classified as weak/medium/strong on the basis of the integration of the respective crosspeaks in the 2D NOESY spectra (τ_m_ = 150 ms) of the complexes and due to the lack of an internal standard of the DNA/ligand relaxation.

No	C-1305 proton	DNA proton	D2L (intensity)	D3L (intensity)
**NOE contacts involving aromatic protons of the ligand**				
1	LH3	B-A5H1′	−	+ (weak)
2	LH3	B-A5H8	+ (medium)	+ (weak)
3	LH3	B-T4H1′	+ (weak)	+ (medium)
4	LH3	B-T4H2′'	+ (strong)	+ (strong)
5	LH3	B-T4H3′	−	+ (weak)
6	LH3	B-T4H6	+ (weak)	+ (weak)
7	LH3	B-T4CH_3_	−	+ (weak)
8	LH4	A-A5H2	−	+ (weak)
9	LH4	B-A5H4′	−	+ (weak)
10	LH4	B-A5H8	−	+ (weak)
11	LH7	A-A5H1′	−	+ (weak)
12	LH7	A-A5H8	+ (weak)	+ (weak)
13	LH7	A-T4H6	+ (medium)	+ (weak)
14	LH7	A-T4CH_3_	+ (medium)	+ (weak)
15	LH7	A-T4H1′	+ (medium)	+ (medium)
16	LH7	A-T4H2′	+ (strong)	+ (strong)^a^
17	LH7	A-T4H2′'	+ (strong)	+ (strong)^a^
18	LH7	A-T4H3′	+ (weak)	−
19	LH9	A-T4CH_3_	+ (strong)	+ (strong)
20	LH10	A-T4CH_3_	+ (weak)	+ (weak)
**NOE contacts involving protons of the aliphatic sidechain of the ligand**				
21	LH15	A-A5H2	+ (weak)	+ (weak)
22	LH15	B-A5H1′	−	+ (medium)
23	LH15	B-A5H2	+ (weak)	+ (weak)
24	LH15	B-A5H4′	−	+ (medium)
25	LH17	A-A5H2	+ (strong)	+ (medium)
26	LH17	B-A5H2	+ (strong)	+ (medium)
27	LH17	B-A5H4′	−	+ (medium)
28	LH18	A-A5H2	+ (strong)	+ (weak)
29	LH18	B-A5H1′	−	+ (medium)
30	LH18	B-A5H2	+ (strong)	+ (medium)
31	LH18	A-A3H2	+ (strong)^b^	−
32	LH18	B-A3H2	+ (strong)^b^	−

^a^These crosspeaks were superimposed.

^b^These crosspeaks were superimposed.

It should be noted that the extensive set of the NOESY contacts observed between the **D2** octamer and the ligand was sufficient to qualitatively reconstruct the intercalation mode, even without the performed in silico refinements. The fact that the aliphatic protons of the ligand exhibited NOE contacts uniquely to the H2 protons of A3 and A5 of the both DNA strands unambiguously placed the aminoaliphatic sidechain inside the minor groove. Regarding the exact positioning of the aromatic moiety, one should notice that the LH3 proton belonging to the one of the peripheral aromatic rings of the ligand interacted uniquely with the protons of one DNA strand (strand **D2L-B**), while the LH7, LH9 and LH10 protons of the opposite aromatic ring gave NOE contacts almost uniquely to the other DNA strand (strand **D2L-A**). Moreover, especially close vicinity was observed between the LH3 and the T4H2″ proton of the strand **D2L-B** and between the LH9 and the T4H2″ proton of the strand **D2L-A**. Such a set of NOEs could be satisfied only while the ligand was placed approximately parallel to the neighboring base pairs and spanned the entire intercalation cavity from the vicinities of the T4 ribose moiety of the one strand to the corresponding ribose moiety of the other.

### 2D NMR studies of the d(CCCTAGGG)2:C-1305 (D3L) complex

Assignment of the ^1^H resonances to the d(CCCTAGGG)_2_:C-1305 (**D3L**) complex was performed in a similar way as it was done for the **D2L** complex. However, in this scenario the **D3L** complex stood out as almost the exclusive (> 90%) DNA spectral form present in the solution, which was quantified upon the comparison of the averaged cytidine H5–H6 and thymidine CH_3_–H6 NOESY cross-peak intensities of the complexed and ligand-free DNA. The intercalation of the ligand into the **D3** duplex once again broke the symmetry of the helix, which manifested in doubling of ^1^H resonances, mostly of the central residues of the complex. Hence, two DNA strands of the complex were *per analogiam* traced separately and named **D3L-A** and **D3L-B**, respectively. Nevertheless, despite the fact that the resonances of the free **D3** duplex were next to non-existent, severe spectral crowding was observed for the C1–C3 and G6–G8 nucleotides of the complex, yielding a more complicated spectral dataset in comparison to the **D2L** study. Still, it was possible to assign all the aromatic base protons of the complex, as well as all the H1′-H3′ deoxyribose resonances (Supplementary Table S2). The H4′ and H5′/H5″ resonances were also partially assigned. The chemical shift changes of the DNA resonances upon complex formation were presented in Fig. 4b in a similar fashion as for the **D2L** complex.

The analysis of the spectral dataset resulted in marking of almost a thirty recorded DNA/ligand intermolecular NOEs, which are listed in Table 1. As it was in case of the **D2L** complex, most of the observed DNA/ligand NOEs were the correlations between the aromatic protons of the ligand and the protons of the T4 and A5 residues of the DNA. On the basis of the aforementioned NOEs, the ligand was unambiguously located—again—at the very centre of the DNA duplex, which was additionally supported by the **D3L-A** and **D3L-B** NOESY-walks. The minor-groove mode of intercalation of the ligand was deduced upon a very similar set of spectral evidences as observed for the **D2L** complex (Table 1).

For the **D3L** complex, it was also possible to assign most of the phosphorus resonances (Supplementary Table S3) through their correlations with sugar protons measured in the HP-COSY spectrum. Most of the backbone phosphorus atoms displayed very limited chemical shift perturbations (< 0.1 ppm) upon C-1305 binding. The only major exceptions from this behavior were provided by the phosphate groups of the two A5 residues, which were downfield shifted by 0.6 and 1.0 ppm in strands A and B, respectively. Such a pattern of chemical shift perturbations throughout the **D3**/**D3L** backbone was another very direct proof of the ligand’s exclusive intercalation into the central T4-A5 dinucleotide step.

### Molecular modelling studies

In order to reveal the atomistic details of the d(CGATATCG)_2_:C-1305 and d(CCCTAGGG)_2_:C-1305 interactions, these systems were subjected to NMR-restrained explicit solvent molecular dynamics simulations. Basing on the pH of the solution and according to previous findings^12^, it was concluded that both the L8-O oxygen atom and the tertiary nitrogen at the end of the ligand’s sidechain (Fig. 1) should be protonated in the refined experimental conditions (II). Hence, two systems were prepared, containing either **D2** or **D3** dsDNA duplexes and C-1305 in a moderate proximity, solvated with water containing appropriate amounts of Na^+^ and Cl^−^. On the basis of Table 1, thirteen (**D2L** complex) and eighteen (**D3L** complex) DNA/ligand distance restraints (DR) were defined, corresponding to the correlations involving the aromatic protons of C-1305. It was decided to not consider the NOEs involving the protons of the ligand’s sidechain, as the preliminary simulations (Supplementary Tables S6 and S7) have demonstrated that not all of them could be satisfied by only one, strictly defined orientation of the sidechain in case of either of the two studied complexes.

The complete and equilibrated systems were then subjected to 1 ns-long restrained molecular dynamics (rMD) simulations. The resulting trajectories revealed that for the both considered sequences C-1305 molecule almost instantly intercalated between T4 and A5 from the minor groove of the DNA. The aliphatic sidechain was maintained in the minor groove, whereas the aromatic system of the ligand slightly protruded from the major groove. Such an arrangement was in perfect agreement with all of the enforced DR. The last frames from these trajectories served as a starting point for two independent 1 µs-long MD simulations for each system. In the first simulation (**D2L-MD1** and **D3L-MD1**), DNA/ligand distance restraints were maintained, while during the second one (**D2L-MD2** and **D3L-MD2**) the respective DR were dropped.

**Figure 5:**
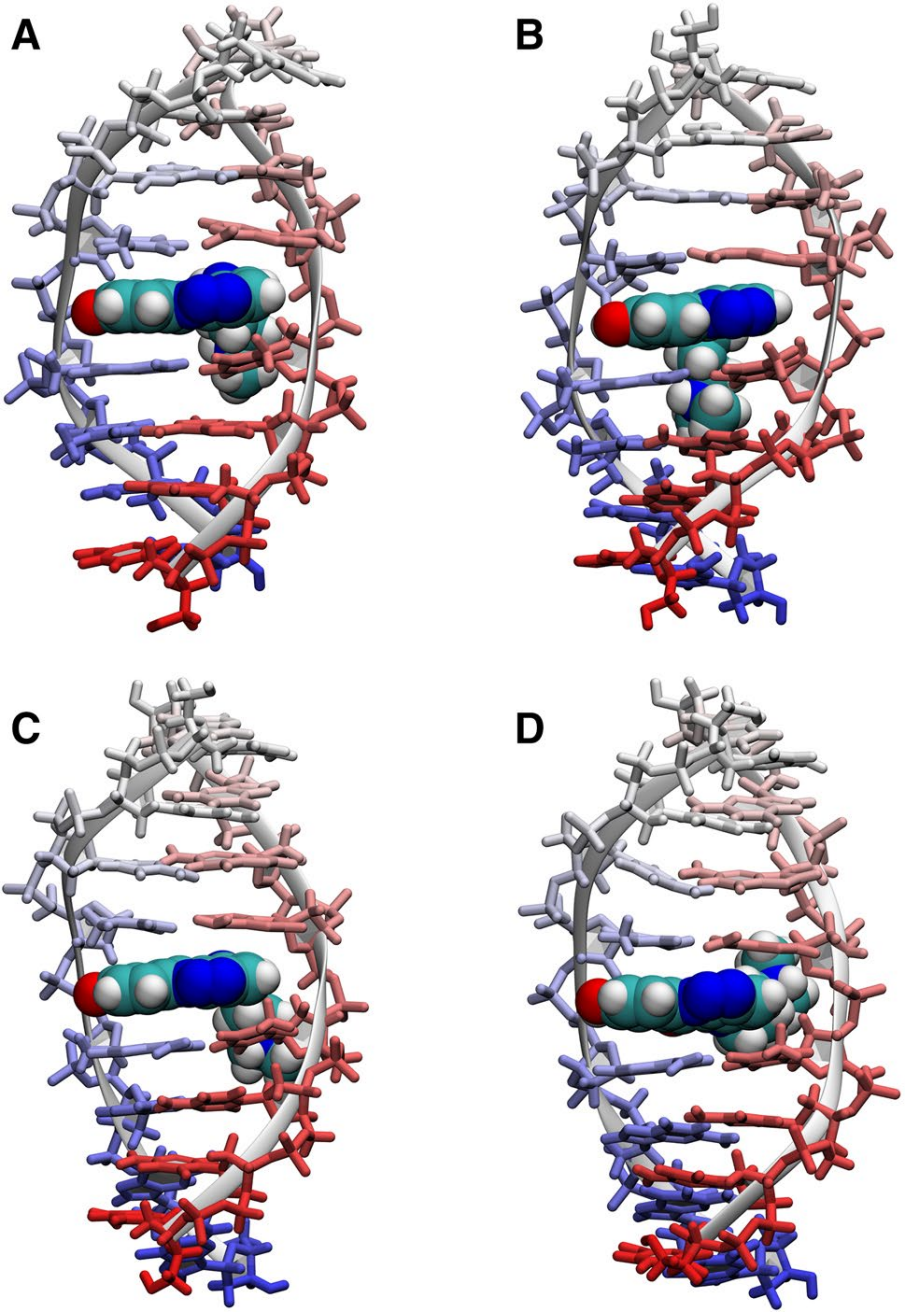
The most representative structures of the **D2L** and **D3L** complexes, obtained as centres of the dominant conformational clusters from the four molecular dynamics simulations: (**A**) **D2L-MD1** (restrained), (**B**) **D2L-MD2** (unrestrained), (**C**) **D3L-MD1** (restrained), (**D**) **D3L-MD2** (unrestrained). For more details on clustering, please consult Supplementary Information. This figure was prepared using VMD 1.9.3^40^, https://www.ks.uiuc.edu/Research/vmd.

All four simulated systems were structurally stable on a simulated timescale. Cluster analysis (Supplementary Figures S4 and S5), performed on the 1 µs trajectories, revealed that the complexes **D2L-MD1** and **D2L-MD2** existed in a similar conformation, classified as the main structural cluster, for 90.6% and 72.1% of the simulation time, respectively (Fig. 5a,b), whereas the systems **D3L-MD1** and **D3L-MD2** exhibited next to identical conformation for 92.2% and 75.6% of the simulation time, respectively (Fig. 5c,d). The structural clusters extracted from NMR-restrained simulations were very well defined, with RMSD of 2.437 Ǻ and 1.652 Å for simulations **D2L-MD1** and **D3L-MD1**, respectively. Moreover, a noticeable fraction of the intercluster structural variability was due to the non-restrained terminal regions of the DNA and when only the central region was considered (residues 3–6 and the ligand), the RMSD decreased to 1.687 Å and 1.229 Å, respectively.

**Figure 6:**
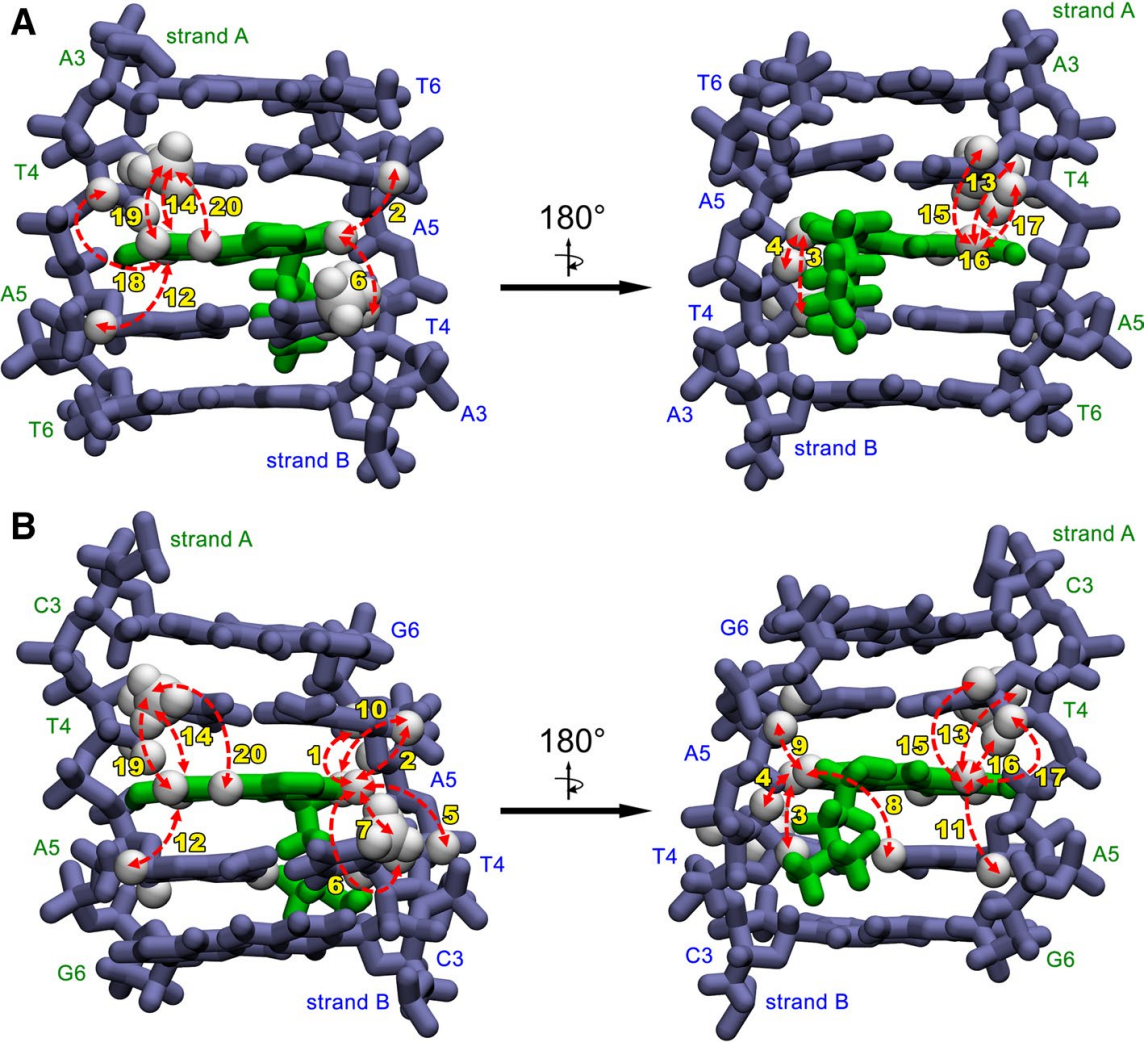
A closer look on the ligand bound at the 5′-TA-3′/5′-TA-3′ intercalation site in (**A**) **D2L** and (**B**) **D3L** complex. Observed NOEs between the aromatic protons of C-1305 and the protons of the DNA are depicted as red, bidirectional arrows. The accompanying numbers correspond to the respective NOE contacts listed in Table 1. This figure was prepared using VMD 1.9.3^40^, https://www.ks.uiuc.edu/Research/vmd.

As it goes for the aromatic system of the ligand, its positioning within the intercalation sites was very well established by the NMR-derived distance restraints. In case of the both examined complexes, the C7–C10 region of the ligand was located between A-T4 and A-A5 nucleotides, whereas the C2–C5 region has settled within the B-T4 and B-A5 bases (Fig. 6a,b). Such a geometry was in perfect agreement with the NOE data reported in Table 1, with just a few observed average NOE violations and no one greater than 0.35 Å (see Table S8 for a detailed comparison of MD-modelled and NOE-derived distances). Such an orientation of the ligand also enabled an occasional formation of the hydrogen bond between the L8-OH group of C-1305 and the A-A5O4′ oxygen atom of the respective deoxyribose moiety^12^. This hydrogen bond was observed in the trajectories of all the conducted MD simulations. Nevertheless, the ligand was still able to slightly rotate within the intercalation sites.

In both simulated systems, the aliphatic sidechain of C-1305 was prone to periodical reorientation within the minor groove, visiting both ends of the DNA helix and reaching up to the third and sixth nucleotides in the sequence. This result explained the observation that the DNA/ligand NOEs involving the protons of the aminoalkyl sidechain couldn’t be satisfied by a single structure in case of either **D2L** complex and **D3L** complex.

For the three-dimensional models of the d(CGATATCG)_2_:C-1305 and d(CCCTAGGG)_2_:C-1305 complexes in PDB format, obtained as centres of the dominant conformational clusters from the restrained **D2L-MD1** and **D3L-MD1** trajectories, please consult Supplementary Data.

### Sequence-specificity studies

The originally selected d(CGATATCG)_2_ (**D2**) duplex contained 4 out of 10 possible dinucleotide steps: CG/CG, GA/TC, AT/AT and TA/TA. In the presence of the TA/TA step in the duplex, the alternative intercalation sites were utterly ignored by the ligand. This result was rather surprising, since, considering the previous studies on the closely related imidazoacridinone C-1311, a symmetrical intercalation into the GA/TC steps^15,16^ was expected. While the experiments reported herein have proven otherwise, in the next step a systematic sequence-specificity study on the triazoloacridinone C-1305 has been initiated, in order to: (1) confirm the binding preference of C-1305 to the TA/TA step in other sequence contexts, (2) examine the CG/CG, GA/TC and AT/AT steps without the presence of the TA/TA step, and finally (3) examine the remaining 6 dinucleotide sequences. Thus, 11 additional dsDNA palindromic duplexes have been engineered in a way that they would contain all 10 possible dinucleotide steps with a varying 5′ and 3′ base-pair neighbourhood. The first four of the designed duplexes (labelled **D3**–**D6**, Table 2) contained TA/TA or AT/AT step at their very centres with the 5′- or 3′-adjacent GGG/CCC triplets. The following seven palindromes (labelled **D7**–**D13**, Table 2) contained TA/TA step not at the centre of their sequences or did not contain this step at all.

**Table 2:** The dinucleotide step preferences of C-1305. The entries in bold represent the intercalation sites unambiguously identified on the basis of the NMR spectra while the remaining ones were inferred through the joint analysis of the entire dataset as described in the “Results” and “Discussion”.

Oligonucleotide codename	Sequence	Relative affinity	Molar ratio of the dsDNA:ligand complex	Dinucleotide steps binding C-1305	Dinucleotide steps missed by C-1305
D1	CCCGGG	Weak	1:1	CG/CG	CC/GG
D2	CGATATCG	Strong	1:1	**TA/TA**	**CG/CG, GA/TC, AT/AT**
D3	CCCTAGGG	Very strong	1:1	**TA/TA**	**CC/GG, CT/AG**
D4	GGGTACCC	Strong	1:1	**TA/TA**	**GG/CC, GT/AC**
D5	CCCATGGG	Medium	1:1	TG/CA	CC/GG, AT/AT
D6	GGGATCCC	None	None	**None**	**GG/CC, GA/TC, AT/AT**
D7	GTACGTAC	Strong	1:2	TA/TA	GT/AC, CG/CG
D8	CTAGCTAG	Strong	1:2	TA/TA	CT/AG, GC/GC
D9	GAACGTTC	Very weak	1:1	CG/CG	GA/TC, AA/TT, AC/GT
D10	CGATGCATCG	Medium-strong	1:1 or 1:2	TG/CA	CG/CG, GA/TC, AT/AT, GC/GC
D11	CGTAGCTACG	Strong	1:2	TA/TA	CG/CG, GT/AC, AG/CT, GC/GC
D12	CTGACGTCAG	Medium	1:2	TG/CA	CT/AG, GA/TC, AC/GT, CG/CG
D13	CTAGCGCTAG	Medium-strong	1:2	TA/TA	CT/AG, AG/CT, GC/GC, CG/CG

The results of the observations on a total of 13 palindromic DNA duplexes interacting with triazoloacridinone C-1305 are listed in Table 2. All the sequences were examined by the means of ^1^H NMR in the previously described, refined experimental conditions (II), in the presence of 1 molar equivalent of the ligand. The ligand intercalation propensity to the different duplexes was estimated based on the ratio of the free and ligand-bound DNA in each sample.

**Figure 7:**
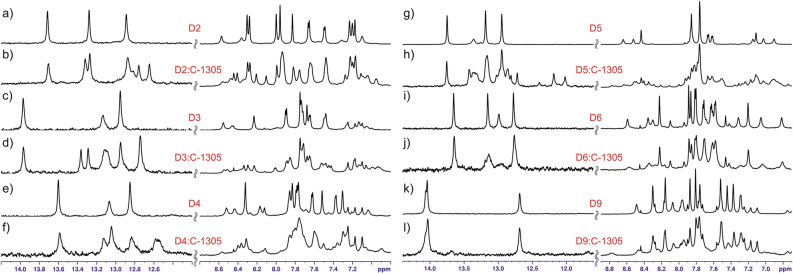
NMR spectra recorded for **D2**–**D6** and **D9** duplexes free in solution (a, c, e, g, i, k) and interacting with the triazoloacridinone C-1305 (b, d, f, h, j, l). In every case, the same concentration of duplex DNA of 0.125 mM was used and the drug was added to achieve 1:1 molar ratio.

Considering the duplexes formed by sequences **D3**–**D6** (Table 2), in case of **D3** and **D4** the ligand intercalated exclusively into the central TA/TA step, producing well-resolved NMR spectra similar to the ones recorded for **D2** (Fig. 7a–f). In a striking contrast, C-1305 exhibited no intercalation into the sequence **D6** at all; only non-specific interactions were observed (Fig. 7i,j). This finding excluded the GG/CC, GA/TC and AT/AT steps from being even low affinity binding sites for C-1305. The elimination of these three steps from a set of the possible binding sites of C-1305 was crucial for the identification of the most plausible binding site(s) in the rest of the studied sequences.

The duplex **D5** interacted with C-1305 with moderate affinity, yet more than one form of the resulting complex was present in the system (Fig. 7g,h). The coexistence of multiple complexed species disabled the possibility of identification of the binding site(s) by 1D NMR. However, after the exclusion of the GG/CC and AT/AT steps (see duplex **D6**) as possible intercalation sites, only the TG/CA step remained as the plausible binding site of C-1305 in case of the duplex **D5**. Yet, this interaction was noticeably weaker than for the TA/TA step. It should be mentioned that the unique intercalation into the TG/CA base step proposed for **D5** is not at odds with the observed coexistence of multiple bound forms. That is due to the fact that the TG/CA step is not present at the centre of the duplex and thus (1) the two different orientations of the ligand (‘up’ and ‘down’) are no longer equivalent and (2) the coexistence of both 1:1 and 1:2 DNA:ligand mol/mol complex stoichiometries is possible. Placing the TG/CA step at the centre of a duplex was not attempted, as this study was limited solely to the palindromic sequences. Whether the GT/AC and AG/CT steps constitute viable intercalation sites remains unsettled for now as they were present in sequences containing the dominating TA/TA site.

At the very beginning of this study, the duplex **D1** (Table 2) exhibited a weak interaction with the ligand, yet it was impossible to identify the binding site of C-1305 due to the severely broadened ^1^H resonances (Supplementary Figure S1). On the basis of the aforementioned **D6**-based GG/CC exclusion, the CG/CG doublet was proposed as a binding site of the ligand in case of **D1**. However, the affinity of C-1305 towards this site cannot be high due to the following observations made for duplex **D9**. The **D9** palindrome contained CG/CG step at the very centre of the sequence, yet it exhibited a barely detectable interaction with triazoloacridinone C-1305 (Fig. 7k,l). As the GA/TC step was previously excluded (see **D6**), in case of **D9** the ligand could have bound to the AA/TT, AC/GT and CG/CG steps. Considering the results obtained for **D1**, the CG/CG step seems to be the suspect, yet this conclusion is unsure. Nevertheless, it is certain that the triazoloacridinone C-1305 does not exhibit considerably high affinity to the AA/TT, AC/GT and CG/CG doublets.

The remaining three, still unsettled dinucleotide steps are the AG/CT, GT/AC and GC/GC. They are present, individually or in-pairs, in the sequences **D7**, **D8** and **D10**–**D13** (Table 2, Supplementary Figure S6). In all of these cases, the binding of the ligand to any of those three doublets was not observed. It must be noted, though, that each one of these six duplexes contained the notably preferred TA/TA or TG/CA steps. Therefore, the resulting conclusion is that the binding of C-1305 to the AG/CT, GT/AC and GC/GC doublets cannot be excluded, yet the ligand’s affinity to any of those three steps has to be incomparably lower than to the TA/TA and TG/CA sequences.

## Discussion

On the basis of the collected experimental data, we have confirmed that triazoloacridinone C-1305 binds into double stranded DNA via intercalation through the minor groove of the helix.

Extensive NMR and MD studies on the d(CGATATCG)_2_:C-1305 (**D2L**) and d(CCCTAGGG)_2_:C-1305 (**D3L**) complexes have revealed that the ligand binds at the very centres of the hosting octamers. Detailed analysis has shown that in both cases the aromatic system of C-1305 slightly protrudes from the major groove, whereas the aliphatic sidechain is located within the minor groove of a DNA duplex. Such an alignment of the ligand was unambiguously defined by two sets of host/guest intermolecular NOEs (Table 1). For instance, for both systems we could observe NOE contacts between the aromatic protons of C-1305 and the T4 methyl groups inside the major groove, whereas the aliphatic sidechain of the ligand exhibited NOE contacts uniquely to the DNA protons located in the minor groove. All the remaining intermolecular NOEs, listed in Table 1, have also strongly supported our resulting conclusion.

Given the established minor-groove-binding mode and the asymmetry of the aromatic region of the ligand, one should notice that C-1305 molecule can enter a given intercalation site assuming one of the two possible orientations of the ring system (“up” or “down”). This should result in two spectral forms of the DNA:ligand adduct per intercalation site. Thus, one should ask the question: why only a single form of the complex was observed for the **D2L** and **D3L** systems? The reason was the fact that the studied triazoloacridinone has intercalated into the very *centres* of the *palindromic* octamers. In such cases, two possible orientations of the ligand’s ring system within a binding site became equivalent. In any other case, the systems’ equilibrium would get more complex, especially if the intercalation of C-1305 would have occurred at multiple, various dinucleotide steps. As a result, we would record barely readable ^1^H NMR spectra consisting of many, overlapping resonances, possibly broadened due to non-specific interactions with the self-associated ligand molecules.

The observed selective binding of C-1305 at the TA/TA step of the originally selected d(CGATATCG)_2_ (**D2**) octamer was a rather unexpected result. We thus continued the research to check whether this was a harbinger of a sequence-specificity of C-1305 to be discovered. Hence, we have examined the interactions of C-1305 with 11 additional short dsDNA palindromes containing all 10 possible dinucleotide steps in varying sequence contexts. This was done in order to provide as deep insight into the potential sequence-specificity of triazoloacridinone C-1305 as possible.

Basing on all the results gathered, we were able to define the C-1305’s dinucleotide step preference in the following order: TA/TA > TG/CA ≫ CG/CG. On the other hand, three other dinucleotide steps: GG/CC, GA/TC and AT/AT were definitively ruled out from the family of the possible binding sites of the triazoloacridinone C-1305. For the remaining four steps: AA/TT, GT/AC, AG/CT and GC/GC, the intercalation was not observed, yet could not be unambiguously excluded based on the available data. It should be noted that all the dinucleotide steps we have observed intercalation into, namely TA/TA, TG/CA and CG/CG, are the 5′-pyrimidine-purine-3′ sequences. Based on the order of affinity towards these steps it can be concluded that triazoloacridinone C-1305 clearly prefers thymidine at the 5′ side of the binding cavity.

The exclusion of the GG/CC step was surprising in the light of the previously reported effective intercalation of C-1305 into the G-triplet-rich oligonucleotides^12^. However, we believe that the previously reported binding of C-1305 into such sequences may be related to the GGG-containing DNA fragments assuming other-than-duplex conformations in a solution. For instance, a G-triplet-rich DNA is likely to form the G-quadruplexes, which were previously demonstrated to be able to effectively bind molecules structurally similar to the triazoloacridinone C-1305. The GGG-containing duplexes used in our study were confirmed in situ to be present uniquely in the duplex form in our refined experimental conditions, while no such test was performed in the previous report^12^.

On the basis of the interactions of C-1305 with the **D2**, **D3** and **D4** duplexes, which contained the AT*AT/AT*AT, CT*AG/CT*AG and GT*AC/GT*AC tetranucleotide steps, respectively (the asterisk stands for the intercalation site), we have observed that while the triazoloacridinone C-1305′s sequence-specificity can be interpreted in terms of dinucleotide steps, the strength of the interaction depends also on the 5′ and 3′ base pairs adjacent to the binding cavity. Previous studies have reported that intercalative binding can induce structural rearrangements in the base pairs separated by one nucleotide from the direct binding site, thus confirming they can play a role in ligand recognition by the DNA^23,24^. Our study revealed that in case of the CT*AG/CT*AG sequence (incorporated in the **D3** duplex) the intercalation of the triazoloacridinone C-1305 was so effective that the system’s equilibrium could be almost completely shifted towards the complex formation, while the narrowness of the ^1^H NMR resonances was still maintained. This was not possible in case of the AT*AT/AT*AT (**D2**) and GT*AC/GT*AC (**D4**) binding regions. The influence of the neighboring base pairs on the C-1305 affinity to the TA/TA step could be explained by the combination of the two effects: (1) the stacking interactions between the 5′- and 3′-adjacent base pairs and the distorted dinucleotide binding cavities, (2) the interactions between the ligand sidechain and the 5′- and 3′-adjacent base pairs. While sidechain/minor-groove interactions were indeed clearly observed during our stereostructural studies, they appear to be rather similar in both the **D2L** and **D3L** complexes. In both cases, the terminal methyl groups of the side chain locate themselves in proximity of the base pairs flanking the intercalation site, and likely undergo regular reorientations from one side of the duplex to the other. The energy of these interactions might still be different in the **D2L** and **D3L** complexes (due to slightly different geometries and charge distributions of the minor grooves), but our current data does not provide definite answers in this matter. Likewise, to reliably evaluate the degree to which the stacking interactions throughout the duplex are differently affected in **D2L** and **D3L**, additional computational studies would be required. Nevertheless, a glance at Fig. 4 allows to conclude that the chemical shift perturbations in **D3L** appear to be mostly confined to the central TA/TA step, while in **D2L** they spread considerably further throughout the duplex. Such a pattern could suggest that only negligible structural changes occur in the neighboring base pairs in **D3L**, while in the case of **D2L** ligand binding induces some geometric rearrangements (likely resulting in stacking distortions) further away from the binding site. Such a comparably less strained bound state for **D3L** might provide a partial explanation for the higher affinity of C-1305 towards **D3**.

The observation of a marked selectivity of C-1305 for TA/TA dinucleotide steps raises the question of the energetic basis for such a high preference towards this specific site. Our NMR-derived atomistic model of the C-1305 molecule bound within a TA/TA dinucleotide step does not reveal any specific interactions (apart from the sequence-independent transient hydrogen bonding between the ligand’s OH group and a deoxyribose moiety). Thus, the reason for the selective intercalation into the TA/TA steps must reside in a favorable energetic balance from some other sources (stacking, hydration etc.). While considering such a balance for the intercalation process at a given site, one has to consider not only the favorable contributions from the newly formed DNA–ligand interactions, but also the energetic penalties for the interactions lost when the two base pairs are separated, i.e., loss of stacking energy between the two base pairs, disruption of specific hydration patterns, etc. While the part of the equation dealing with the DNA–drug interactions is of course ligand-dependent, the penalty for the separation of a given dinucleotide step is, on the other hand, an intrinsic property of the dsDNA itself and, as such, has been extensively studied. Multiple studies aimed at the understanding of the DNA thermodynamics have formulated the findings in terms of intrinsic stacking energies at different dinucleotide steps, as gathered for example by SantaLucia^25^. However, perhaps the most direct estimation of a dinucleotide step separation energy comes from the study of the stacked-unstacked equilibria in the nicked DNA molecules of different sequences^26^. Interestingly, the energetic penalty of the separation of the TA/TA step is the lowest one among all the possible dinucleotide steps according to both SantaLucia^25^ and Protozanova et al.^26^. Thus, it may appear that for the intercalators with the relatively low differences in the dsDNA:ligand interaction energies at different dinucleotide steps, the TA/TA step should stand out as the default binding site. Whether the TA/TA preference of C-1305 is actually decided by this mechanism is difficult to prove with the current data and some additional research is needed, aiming at evaluation of dsDNA:C-1305 stacking interactions at various dinucleotide steps. Nevertheless, an additional hint in favor of such an interpretation is provided by the fact that TG/CA step, the other site preferred by C-1305, is also the second most easily separated one according to Protozanova et al.^26^. Regardless whether our hypothesis is true, the striking difference in the ligand’s affinity to the TA/TA and AT/AT steps is evident (stacking energies of 0.2 kcal/mol and 1.3 kcal/mol, respectively^26^). Such a preference was actually observed for several other intercalators, also some structurally unrelated to C-1305. For instance, intercalation into the very centre of the d(CGTACG)_2_ duplex was previously reported for ACRAMTU, another acridine-based dsDNA topoisomerase II poison^27^. The Authors established the TA/TA, CG/CG and GA/TC as the preferred binding sites of the drug, while the compound intercalated most effectively into the d(TATATATA)_2_ octamer. In a striking contrast, the d(AGGGCCCT)_2_ and d(AAAATTTT)_2_ duplexes were proven to be the disfavored recipients of ACRAMTU^27^. In another study^28^, the d(CCGGTACCGG)_2_ and d(CCGGATCCGG)_2_ DNA duplexes were crystalized in the presence of λ-[Ru(phen)_2_dppz]^2+^. In case of the d(CCGGTACCGG)_2_ sequence deep intercalation into the TA/TA step was observed, while the inverted AT/AT step, present in the second duplex, was utterly ignored^28^. The Authors of this report explained this selectivity by excellent ligand-DNA shape-complementarity at the TA/TA step. Recently, Cu-Oda was demonstrated to be able to distinguish between the TA/TA and AT/AT steps. The copper complex was proven to effectively intercalate into the TA/TA step, whereas it induced the transition from B-DNA to Z-DNA while bound to the AT/AT dinucleotide sequence^29^.

The intercalation of a drug molecule into dsDNA is a complex process involving a delicate balance between the energetic contributions from stacking interactions lost and formed, as well as from specific hydrogen bonding interactions. The net effect of all these factors dictates the sequence specificity of a given intercalator. For the triazoloacridinone C-1305 studied here we have found a marked preference for the interaction with TA/TA step, characterized by very low intrinsic stacking energy. As the interaction lacks specific hydrogen bonding, it seems reasonable to assume that this property has a substantial contribution into determining the binding specificity. Such an interpretation might hold true for other intercalators, selecting the TA/TA step without forming specific hydrogen bonding at this site.

## Materials and methods

### NMR sample preparation

All the chemically synthesized oligonucleotides used in this study were purchased from Sigma-Aldrich. Before the NMR measurements, each purchased oligo was further purified by precipitation from acetone containing 1% NaClO_4_. This process served to remove an impurity giving rise to very strong signals in the proton NMR spectra at around 1.28, 1.99, 3.21 and 8.60 ppm (triethylamine-acetate, used by the oligo supplier during HPLC purification). Thanks to their palindromic nature, each oligo used in this study is fully self-complementary and thus the preparation of the duplex samples consisted simply of dissolving the purified material in an appropriate buffer. A range of buffer compositions (buffering agent, pH, NaCl concentration) were tested throughout this study in a pursuit to optimize the quality of the measured NMR spectra. The two most relevant conditions (see “Results”) were: (I) 10 mM phosphate buffer of pH 7.5, containing 150 mM NaCl and (II) 2.5 mM cacodylate buffer of pH 5.0, containing 10 mM NaCl. To prepare the samples of the dsDNA:C1305 intercalation complexes, the ligand was added to the pre-mixed NMR sample from a concentrated stock solution in water, to reach to duplex:ligand molar ratio of 0.5, 1.0, 1.25 or 2.0, depending on the sample.

### NMR spectra

All NMR spectra were collected using a 700 MHz Bruker Avance III HD spectrometer, equipped with a QCI CryoProbe. After two 8 bp DNA duplexes forming a single, well-defined complexes with C1305 were identified (see “Results”), a set of 2D spectra was recorded for resonance assignment for each of the free duplexes. It comprised the NOESY (150 ms mixing time) and TOCSY (60 ms spin-lock time) spectra measured at 5 °C in 90% H_2_O/10% D_2_O, as well as, the NOESY (150 and 400 mixing time), HC-HSQC, HP-COSY and DQF-COSY spectra acquired in 100% D_2_O at 25 °C. These spectra were recorded in conditions (I). The resonance assignment process itself was performed using standard approaches^21^. In order to obtain the reference data for the ligand, NOESY (150 mixing time) and TOCSY (60 ms spin-lock time) spectra were recorded for this compound at 5 °C in 90% H_2_O/10% D_2_O in conditions (I) and (II). For the assignment of the dsDNA–C1305 complexes and for the identification of the DNA–ligand cross-peaks, the NOESY (150 ms mixing time), TOCSY (60 ms spin-lock time) and HC-HSQC spectra were recorded for the complexes at 5 °C, in both 90% H_2_O/10% D_2_O and 100% D_2_O in conditions (II). HP-COSY spectra were also recorded for the two complexes, but only in the case of **D3L** a good quality spectrum was observed as for **D2L** the resonances turned out to be too broad to provide enough signal.

The sets of spectra originally collected to assign the free duplexes were recorded in somewhat different experimental conditions (I) than the spectra of the dsDNA:C-1305 complexes (II). Thus, in order to extract the most meaningful chemical shift differences due to complex formation, the following procedures were applied to reassign the free duplexes in conditions (II). The **D2** duplex, which coexisted in a significant quantity with the **D2L** complex, was reassigned directly from the spectra recorded for the complex. For the **D3** duplex, additional NOESY spectra were recorded at 5 °C in conditions (II), in both 90% H_2_O/10% D_2_O and 100% D_2_O. Using these spectra, the assignments made previously in conditions (I) were easily transferable to conditions (II).

Fragments of TOCSY and NOESY spectra, displaying correlations essential for assignment of ligand’s resonances and identification of intermolecular DNA:C-1305 interactions in **D2L** and **D3L** complexes have been presented in Supplementary Information as Figures S7–S14.

### Molecular modelling

Molecular dynamics (MD) simulations were performed for the d(CGATATCG)_2_:C-1305 and d(CCCTAGGG)_2_:C-1305 intercalation complexes explicitly solvated in cubic boxes, with 4,497 and 4,502 TIP3P water molecules, respectively, at 0.15 M concentration of NaCl. The force field parameters for the DNA octamers were taken from the latest iteration of CHARMM36 nucleic acid force field^30^. The parameters for C-1305 were taken from the latest version of CHARMM36 Generalized Force Field (CGenFF)^31^, whereas the partial atomic charges of the ligand were calculated ab initio using GAUSSIAN09 software on the MP2/6-31G* level of theory^32^. All energy minimizations and MD simulations were carried out using GROMACS 2018.2^33^. All the MD simulations were conducted using the leapfrog scheme with a time step of 2 fs. The particle mesh Ewald technique with a cutoff of 1 nm and grid spacing of approx. 0.1 nm was used to evaluate electrostatic forces^34^. The van der Waals interactions were calculated using Lennard–Jones potential with a cutoff of 1 nm. The simulations were conducted at a constant temperature of 278 K and at a constant pressure of 1 bar, using the weak coupling method^35^.

Preparation of the molecular models of the dsDNA:ligand complexes was identical for the both studied sequences and was executed as follows. After obtaining an initial B-form of DNA structure from X3DNA 2.3^36^, one C-1305 molecule with a protonated tertiary nitrogen at the end of the sidechain was placed in a moderate proximity of a DNA duplex using VMD software. After the appropriate energy minimization and 100 ns of MD-based initial equilibration with position restraints set on DNA and ligand molecule, the system was simulated for 1 ns. During this run, 13 (**D2L** complex) and 18 (**D3L** complex) distance restraints corresponding to the NOE contacts between the aromatic protons of the ligand and the protons of the DNA were applied (Table 1). This was done using the GROMACS implementation of the restraining potential which adds a quadratic penalty to the potential when a distance exceeds a lower or upper threshold. The same force constants of 1,000 kJ mol^−1^ nm^−2^ were used for all restrained distances. The above described simulation resulted in a DR-driven intercalation of C-1305 molecule into the 5′-TA-3′/5′-TA-3′ site from the minor groove of the DNA duplex. Afterwards, the final frame from the resulting trajectory was extracted. This frame was set as a starting point for the two independent 1 µs-long MD simulations described below. Each of them was preceded by 100 ns of further equilibration with position restraints set on DNA and ligand molecules.

During the first run, designated as **D2L-MD1** and **D3L-MD1** in the main text, respectively, the systems were subjects to the aforementioned 13 (**D2L**) and 18 (**D3L**) distance restraints derived from the NOESY experiment of τ_m_ = 150 ms and another 14 (**D2L**) and 16 (**D3L**) distance restraints, strengthening the hydrogen bonds in Watson–Crick base pairs. It was decided to stabilize all the base pairs except the terminal GC pairs, since the G8 imino proton resonance was not observed in the ^1^H NMR spectra of the both **D2L** and **D3L** complexes.

During the second run, designated as **D2L-MD2** and **D3L-MD2** in the main text, the systems were subjects only to the 14/16 distance restraints strengthening the hydrogen bonds in Watson–Crick base pairs. This was done in a similar way as it was described above for the **D2L-MD1** and **D3L-MD1** simulations.

For more details on distance restraints, please consult Supplementary Data (Supplementary Tables S4 and S5).

Cluster analysis was performed using the Daura method^37^.

## Supplementary information

Supplementary Information 1.

Supplementary Information 2.

Supplementary Information 3.

## Data Availability

Most of the data generated or analyzed during this study are included in this published article (and its Supplementary Information files). The remaining datasets generated and analyzed during the current study, i.e. full NMR spectra and MD trajectories, are available from the corresponding author on reasonable request.
