# Supplementary material for: A strong preference for the TA/TA dinucleotide step discovered for an acridine-based, potent antitumor dsDNA intercalator, C-1305: NMR-driven structural and sequence-specificity studies
Source: Sci Rep. 2020 Jul 16;10:11697. doi: 10.1038/s41598-020-68609-8 (PMC7366671; doi:10.1038/s41598-020-68609-8)
Supplement: Supplementary file 3 — Supplementary Information 3. [file 41598_2020_68609_MOESM3_ESM.doc]

**A strong preference for the TA/TA dinucleotide step discovered for an acridine-based, potent antitumor dsDNA intercalator, C-1305. NMR-driven structural and sequence-specificity studies**

**Tomasz Laskowski^1,†^,* & Witold Andrałojć^2,†^,**, Jakub Grynda^1^, Paulina Gwarda^1^, Jan Mazerski^1^, and Zofia Gdaniec^2^**

^1^ Department of Pharmaceutical Technology and Biochemistry, Faculty of Chemistry, Gdańsk University of Technology, Gdańsk, Gabriela Narutowicza Str. 11/12, 80-233, Poland

^2^ Institute of Bioorganic Chemistry, Polish Academy of Sciences, Poznań, Zygmunta Noskowskiego Str. 12/14, 61-704, Poland

* [tomasz.laskowski@pg.edu.pl](mailto:tomasz.laskowski@pg.edu.pl)

** [wandralojc@ibch.poznan.pl](mailto:wandralojc@ibch.poznan.pl)

^†^ The authors wish it to be known that, in their opinion, the first two authors should be regarded as joint First Authors.

**SUPPLEMENTARY DATA**


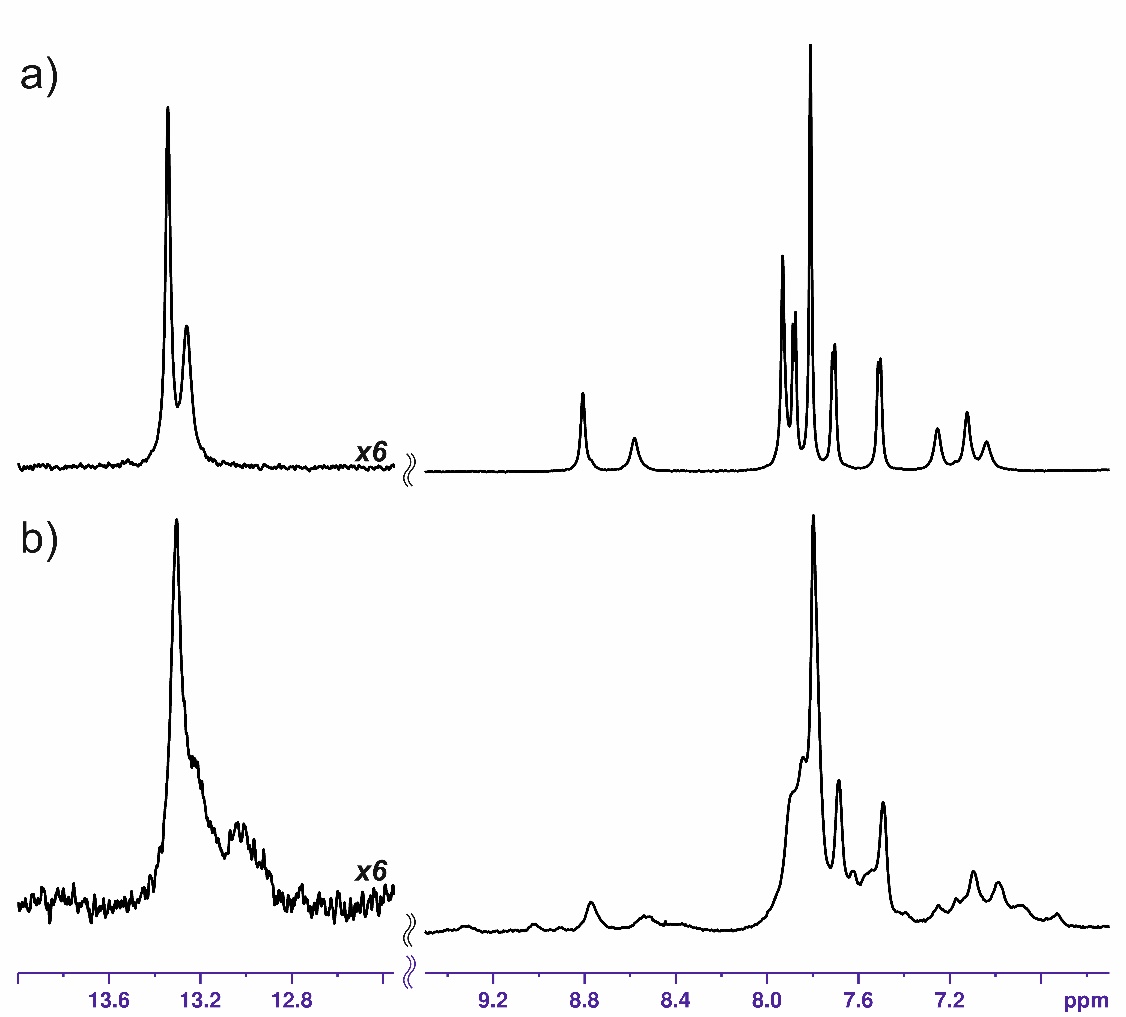


**Figure S1.** NMR studies of the d(CCCGGG)_2_:C-1305 complex: a) reference spectrum of the d(CCCGGG)_2_ (**D1**) duplex, b) d(CCCGGG)_2_ duplex in the presence of 1 molar equivalent of C-1305. A non-specific DNA/ligand interaction is dominating over the drug’s intercalation, resulting in significant broadening of the resonance lines.

**On the optimization of the experimental conditions for the dsDNA:C-1305 complex formation**

In the ***Optimization of the experimental conditions*** paragraph within the ***Results*** section of the main article, we have stated that at the higher concentrations of the d(CGATATCG)_2_:C-1305 complex components within the NMR tube (c > 1 mM), a significant broadening of the proton resonances could be observed while the complex was formed in a widely used, physiologically-relevant experimental conditions, namely: 10 mM phosphate buffer, pH = 7.0, 150 mM NaCl. Surprisingly, higher concentrations also slightly shifted the system’s equilibrium towards the complex dissociation. We believe that this observation, along with the process of the optimization of the experimental conditions, require some additional discussion.

The results obtained in physiologically-relevant conditions can be explained by the strong self-association tendency of C-1305. Self-association of C-1305 in water had been proven experimentally before by our group using UV-VIS and NMR studies and was shown to occur already at very low concentrations (Figure S3). Thus, the aggregation of the ligand should be considered as a competitive process with respect to its intercalation into dsDNA, which explains the observed equilibrium’s shift away from the intercalation complex at higher ligand concentrations. The accompanying broadening of the ^1^H resonances could in turn be assigned to non-specific interactions of the positively charged aggregated ligand molecules with both complexed and non-complexed forms of the DNA duplex. Moreover, by investigating a set of samples in differing conditions, we have also discovered that the efficiency of the self-association of C-1305 depends not only on the concentration of the compound, but also on the pH of the water solution and its ionic strength (data not shown). In a series of simple 1D NMR experiments we have found that at a given concentration of the ligand, lowering the ionic strength and the pH of the buffer significantly shifts the system’s equilibrium towards the monomeric form of C-1305. Therefore, we decided to alter the experimental conditions of the complex formation in a way that would keep the oligomer’s ability to form a DNA duplex, while the C-1305’s preference for self-association would be significantly muted. We traced the progress of our optimization process by observing the imino proton resonances (1) and the two singlet resonances at δ ~ 3 ppm (2). While (1) appear in a range of 12-14 ppm, they mark the presence of a double stranded DNA in a solution, which means that the experimental conditions do not prevent the duplex formation. On the other hand, (2) correspond to the methyl groups of the ligand. The one with the higher resonance frequency can be assigned to the C-1305 intercalated into dsDNA, whereas the singlet of the lower resonance frequency corresponds to the free ligand molecules, which exist mostly in an aggregated form in solution. Tracing the relative intensities of these two singlets enabled an on-the-fly evaluation of the percentage of C-1305 molecules that have intercalated into dsDNA (Figure S4). Therefore, we could monitor what fraction of them was present in a self-associated form in a solution, broadening the ^1^H resonances of a whole system.

Ultimately, the optimal experimental conditions for the d(CGATATCG)_2_:C-1305 complex formation, to a large extent inhibiting the self-association process of the ligand, were identified as: 2.5 mM cacodylate buffer, pH = 5.0, 10 mM NaCl.


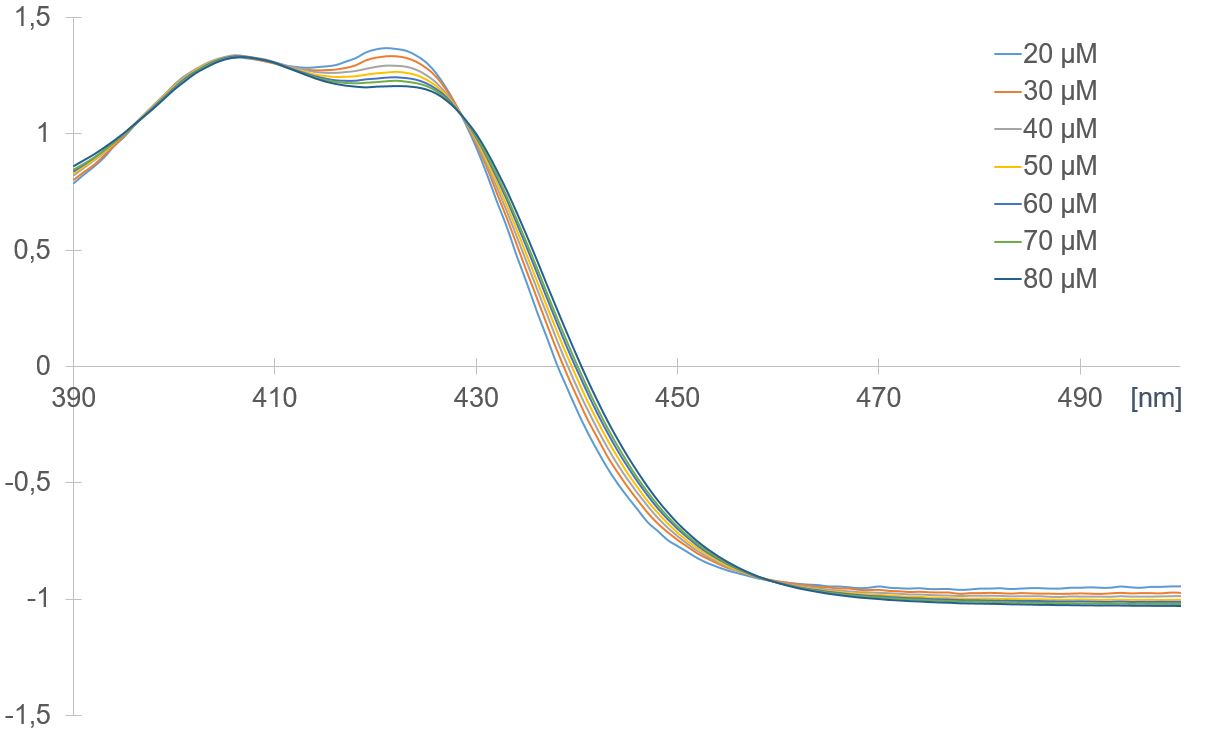


a)


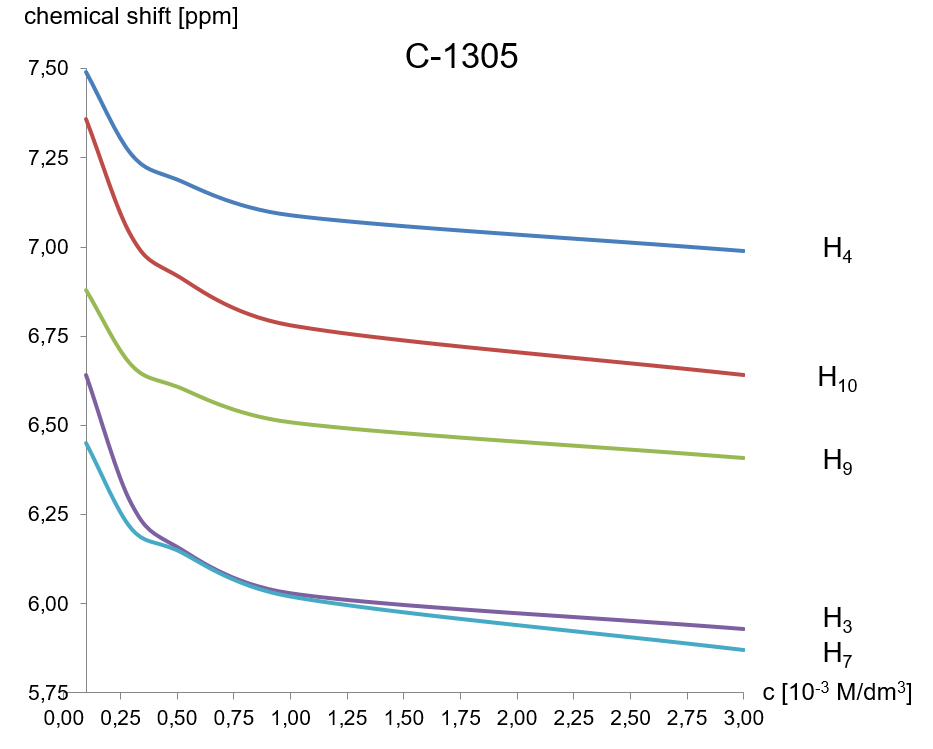


b)

**Figure S2.** UV-VIS (a) and NMR (b) studies on the self-association of C-1305 in water solutions, conditions: 10 mM phosphate buffer, pH = 7.4, 150 mM NaCl, 50 µM EDTA. Image (a) represents normalized molar extinction coefficient for a series of C-1305 concentrations. It can be seen that increasing the compound’s concentration results in decreasing of ~425 nm maximum’s intensity, as well as in its bathochromic shift, which is a direct result of the aggregation of the compound. Image (b) represents changes in the ^1^H chemical shifts (δ, ppm) of the aromatic protons of C-1305 upon decreasing the compound’s concentration. Significant increase of δ at the low concentrations region results from shifting the system’s equilibrium towards the monomeric form of C-1305.


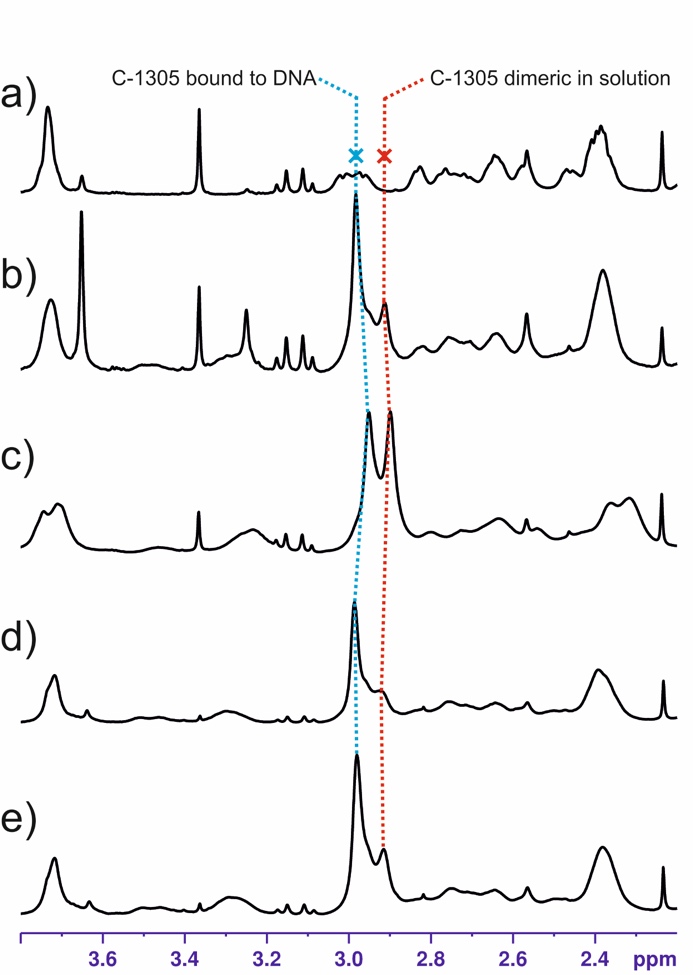
**Figure S3.** Tracing of the ligand behavior in a solution during the optimization of the experimental conditions for the d(CGATATCG)_2_:C-1305 complex formation: a) uncomplexed DNA duplex, b) DNA:ligand 1:1 mol/mol in physiologically-relevant conditions, c_DNA_ = 125 µM, c) DNA:ligand 1:1 mol/mol in physiologically-relevant conditions, c_DNA_ = 1.25 mM, d) DNA:ligand 1:1 mol/mol in optimized conditions, c_DNA_ = 1.25 mM, e) DNA:ligand 1:1.25 mol/mol in optimized conditions, c_DNA_ = 1.25 mM. The dotted lines mark position and intensity of the resonances of the methyl groups of C-1305 bound to DNA (blue) and aggregated in solution (red).


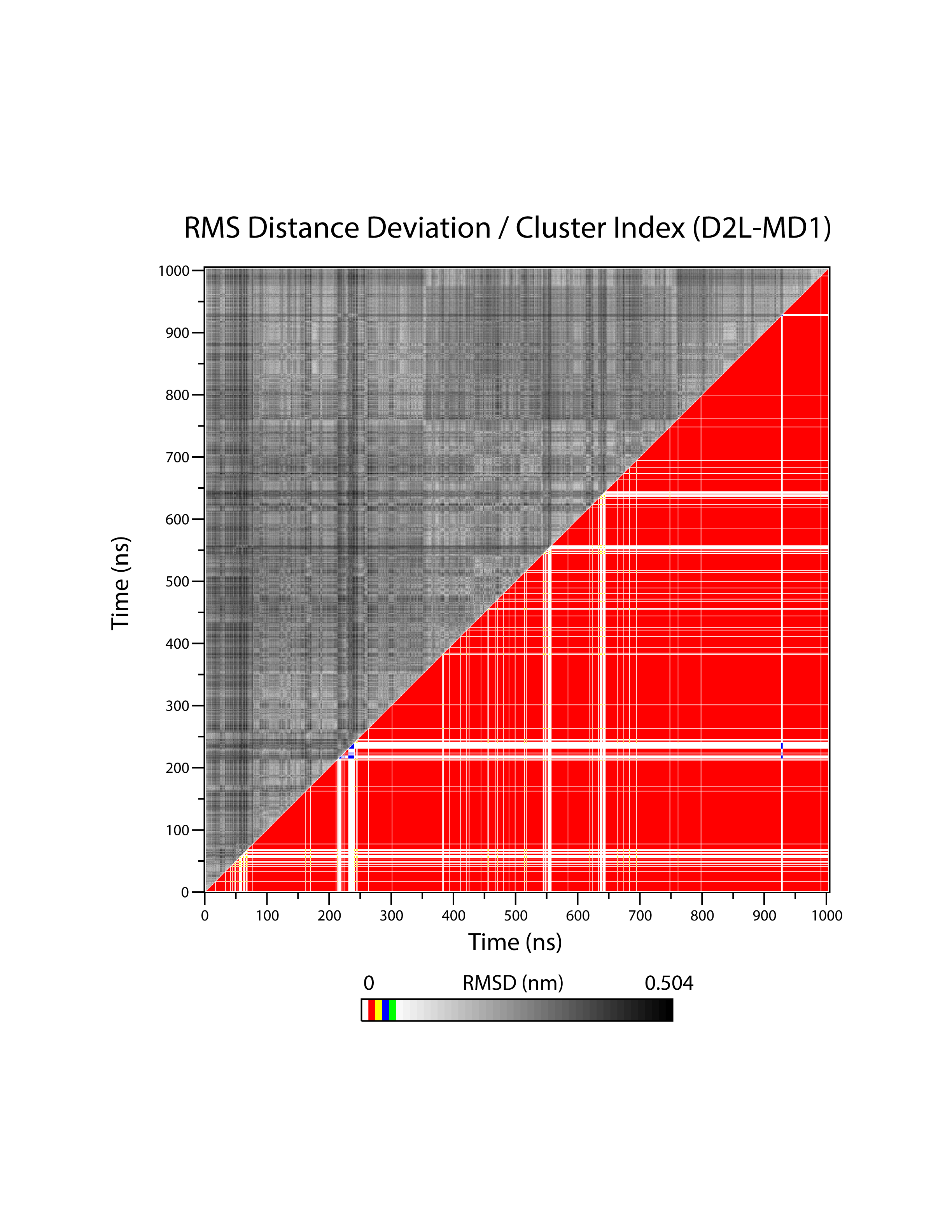


**Figure S4.** Cluster analysis of the restrained MD simulation of the d(CGATATCG)_2_:C-1305 complex (**D2L-MD1**). Red color depicts the conformations belonging to the dominant conformational cluster.


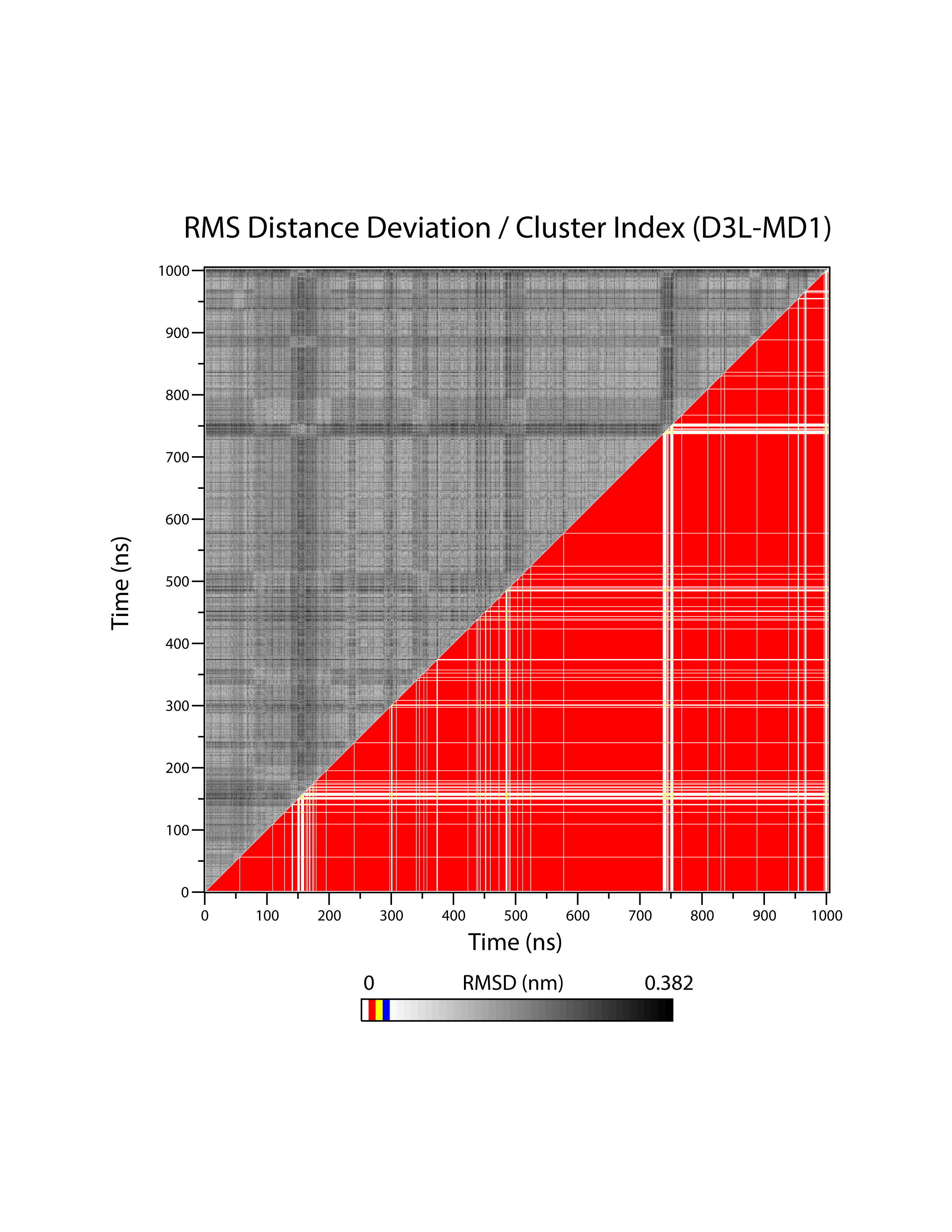


**Figure S5.** Cluster analysis of the restrained MD simulation of the d(CCCTAGGG)_2_:C-1305 complex (**D3L-MD1**). Red color depicts the conformations belonging to the dominant conformational cluster.


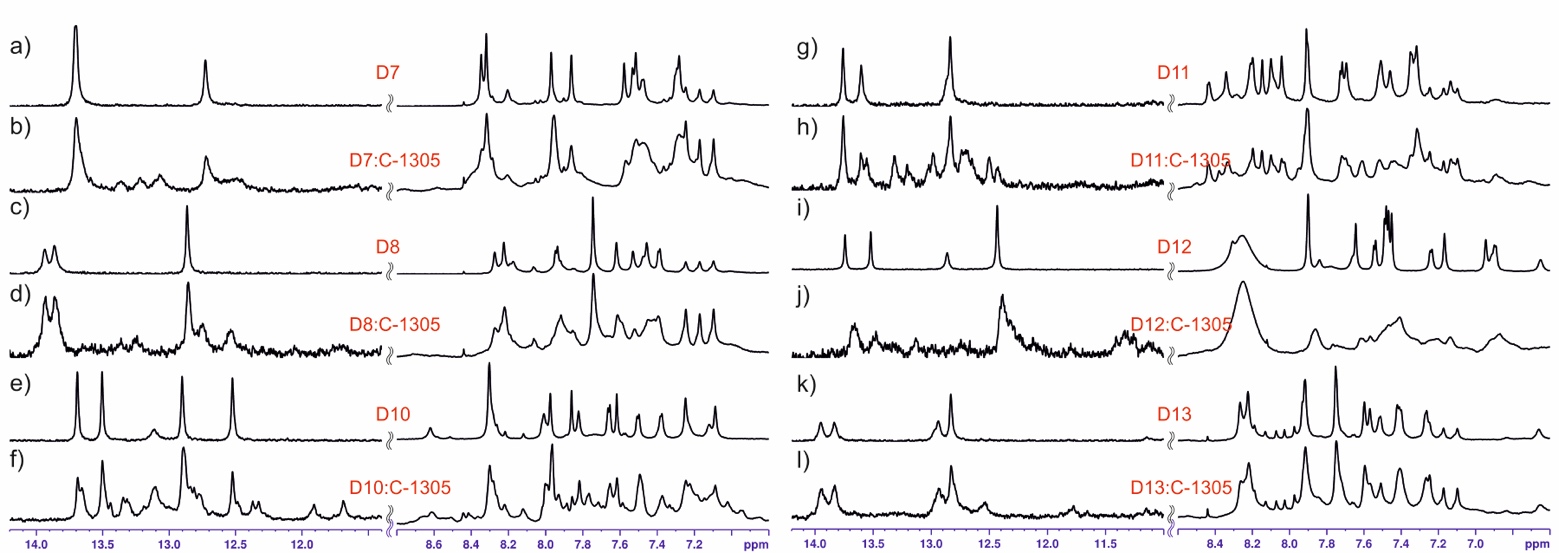


**Figure S6.** NMR spectra recorded for **D7**, **D8** and **D10**-**D13** duplexes free in solution (a, c, e, g, i, k) and interacting with the triazoloacridinone C-1305 (b, d, f, h, j, l). In every case, the same concentration of duplex DNA of 0.125 mM was used and the drug was added to achieve 1:1 molar ratio


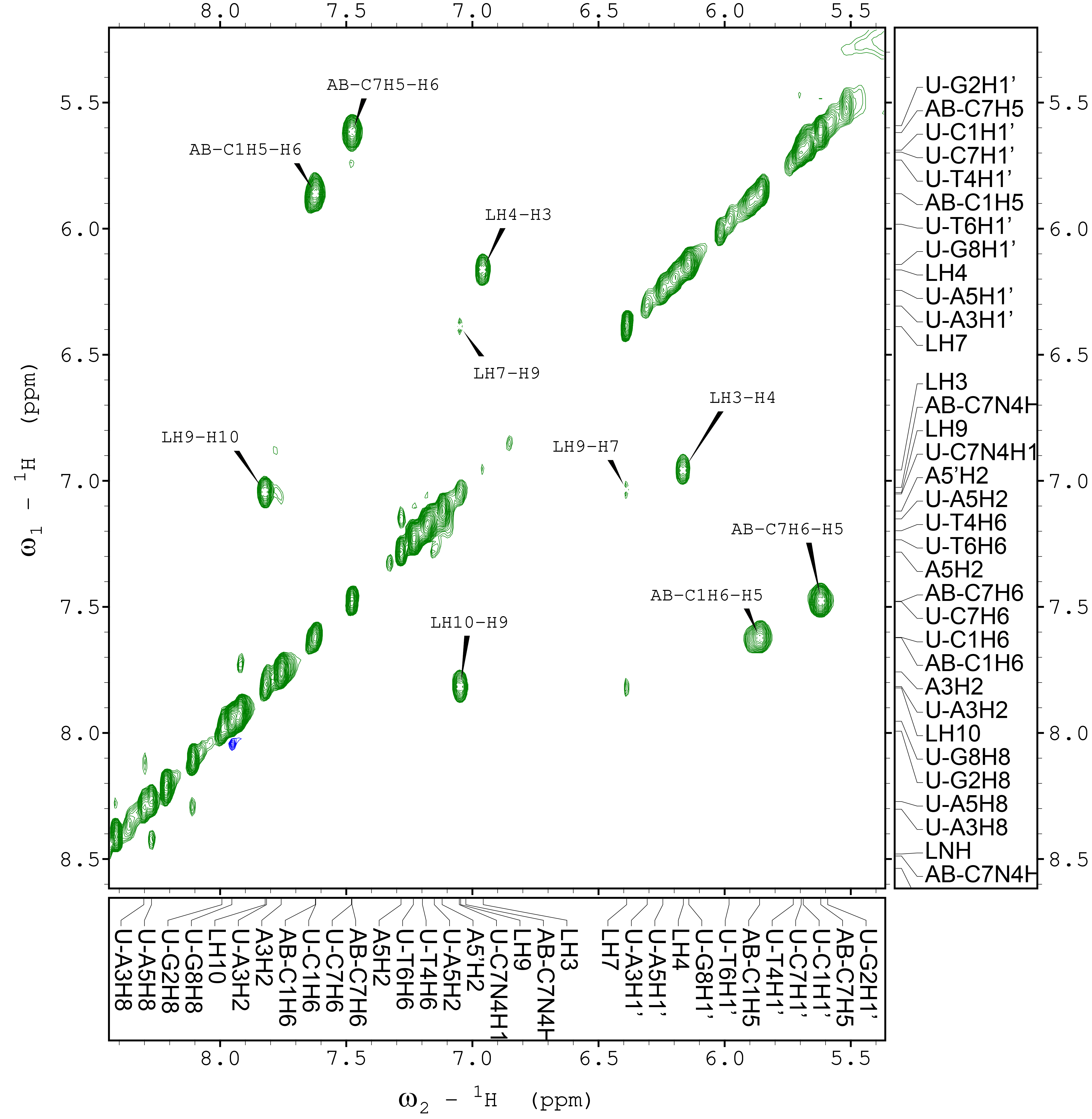


**Figure S7.** Fragment of the TOCSY spectrum of the d(CGATATCG)_2_:C-1305 complex, displaying correlations between the aromatic protons of the ligand. Spectrum was recorded in H_2_O/D_2_O 9:1 v/v.


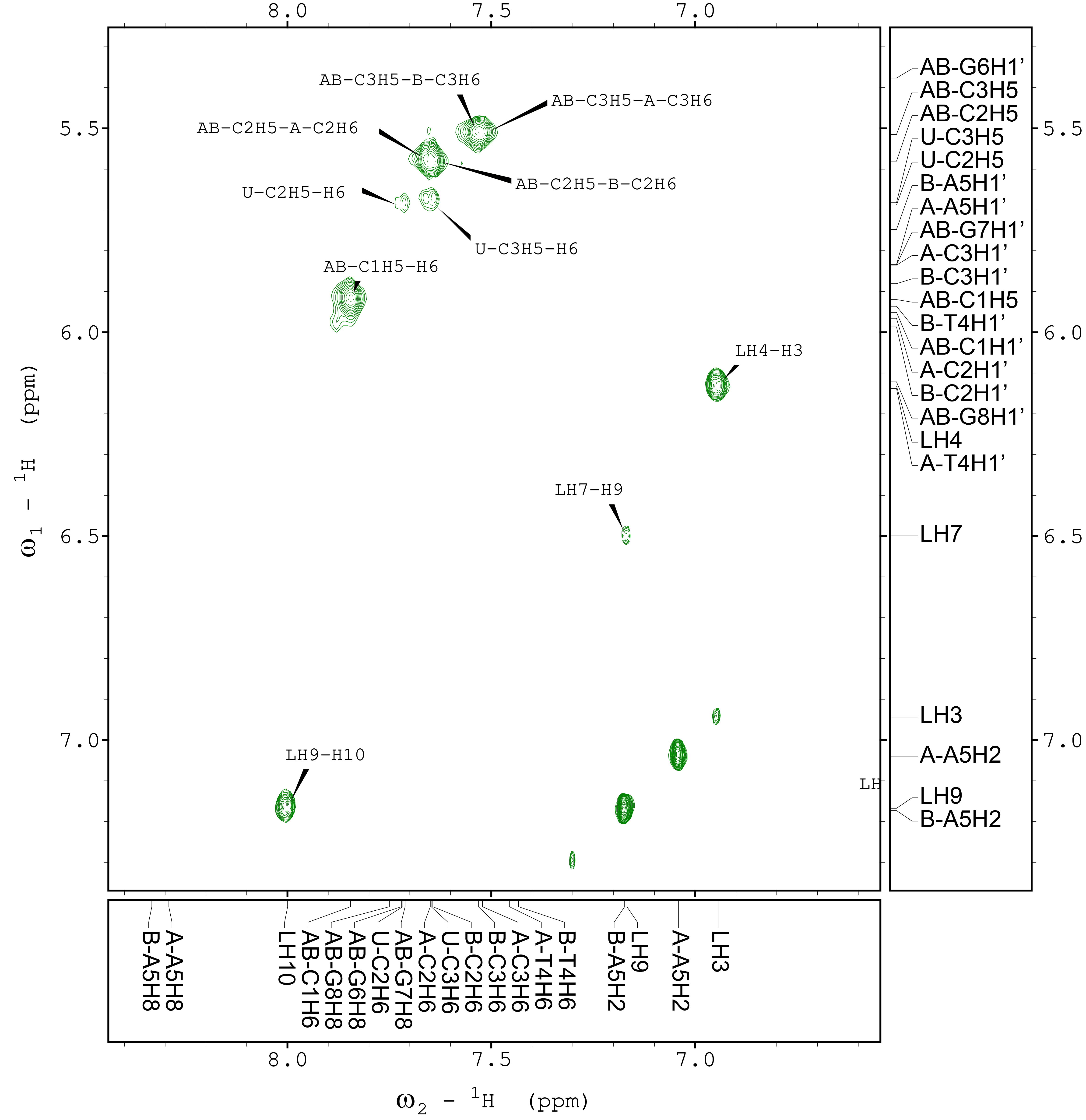


**Figure S8.** Fragment of the TOCSY spectrum of the d(CCCTAGGG)_2_:C-1305 complex, displaying correlations between the aromatic protons of the ligand. Spectrum was recorded in D_2_O.


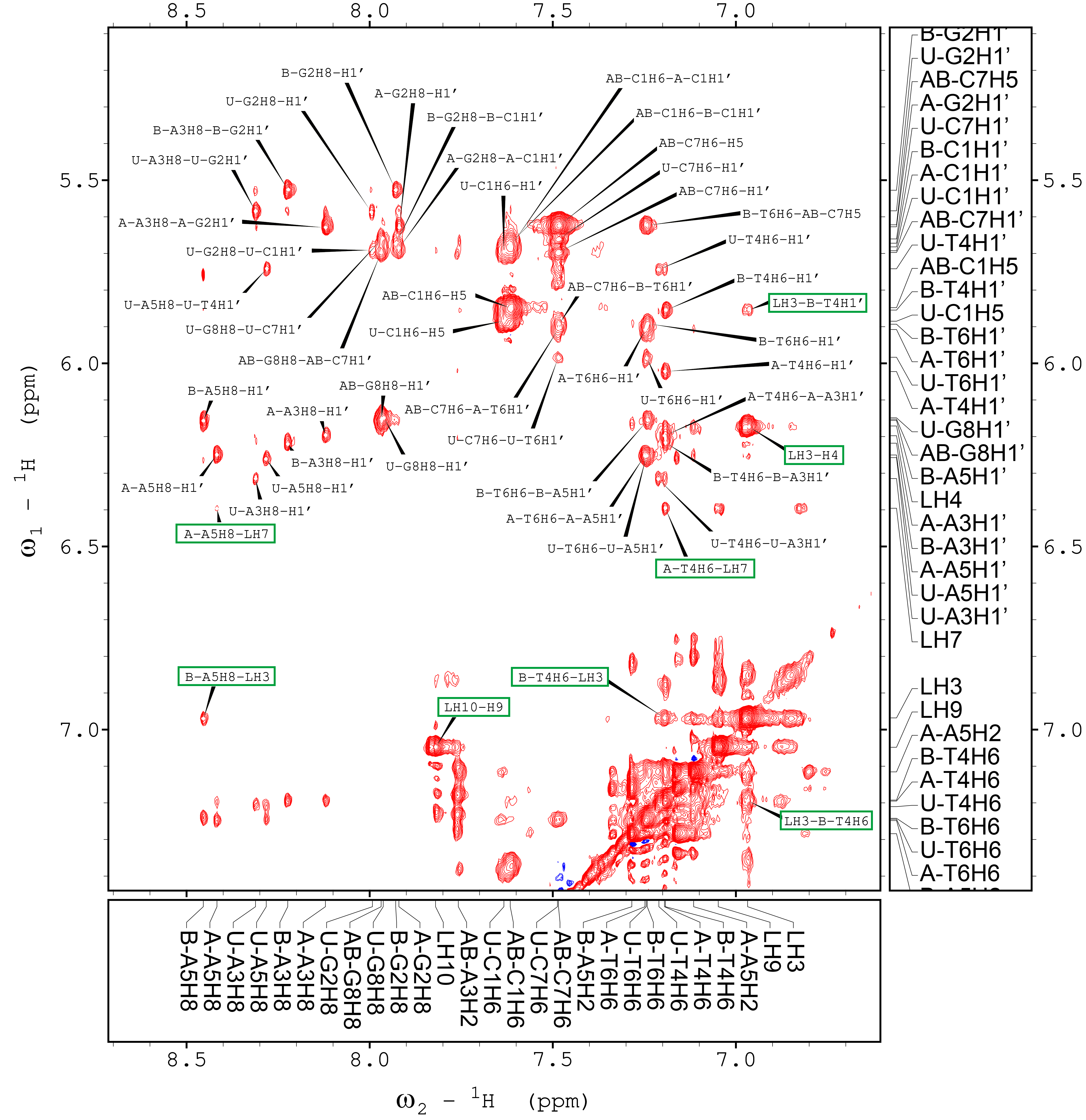


**Figure S9.** Fragment of the NOESY spectrum of the d(CGATATCG)_2_:C-1305 complex, displaying correlations incorporating aromatic protons of the system. NOEs involving protons of the ligand have been highlighted by green rectangles. Spectrum was recorded in D_2_O.


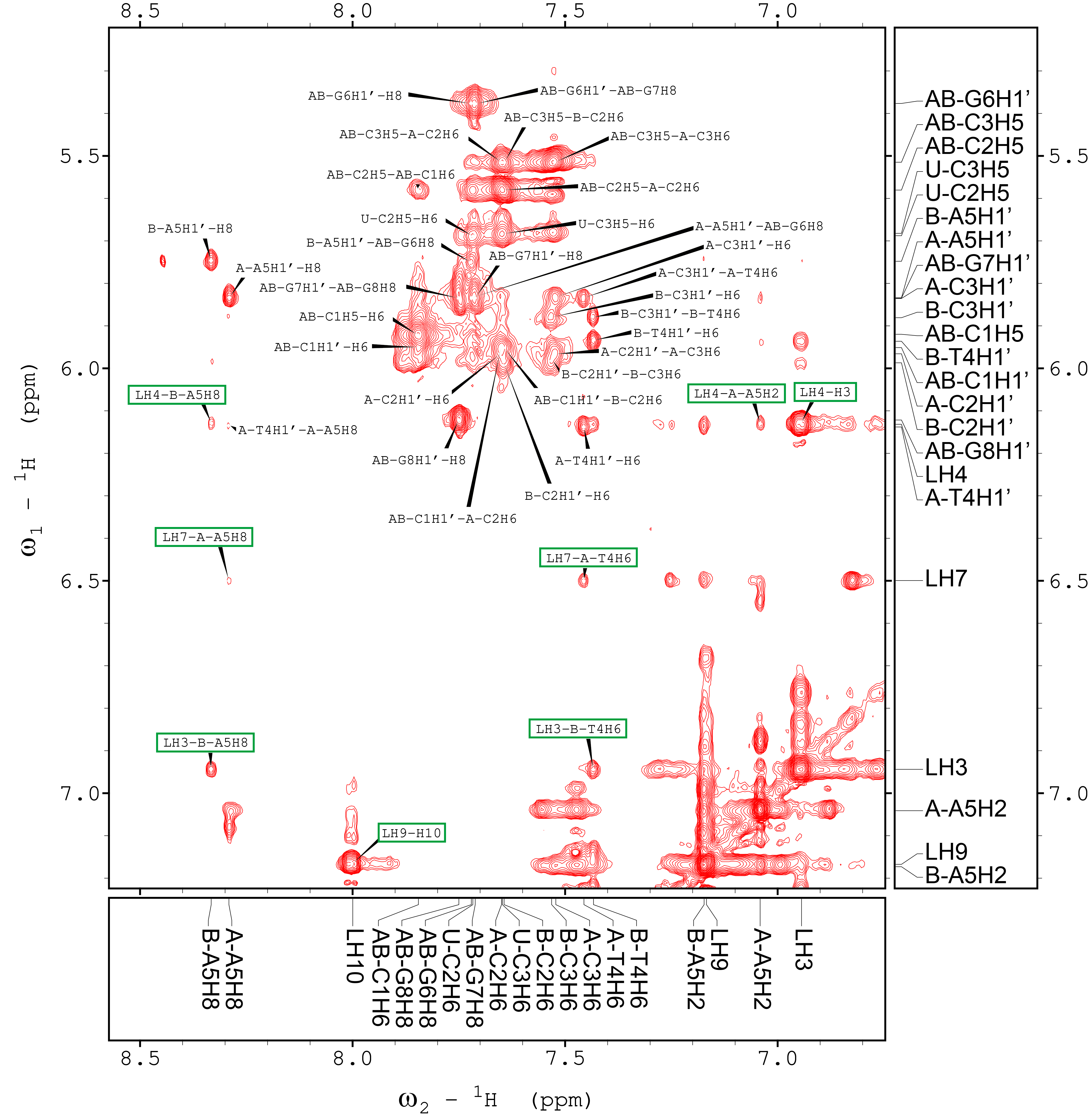


**Figure S10.** Fragment of the NOESY spectrum of the d(CCCTAGGG)_2_:C-1305 complex, displaying correlations incorporating aromatic protons of the system. NOEs involving protons of the ligand have been highlighted by green rectangles. Spectrum was recorded in D_2_O.


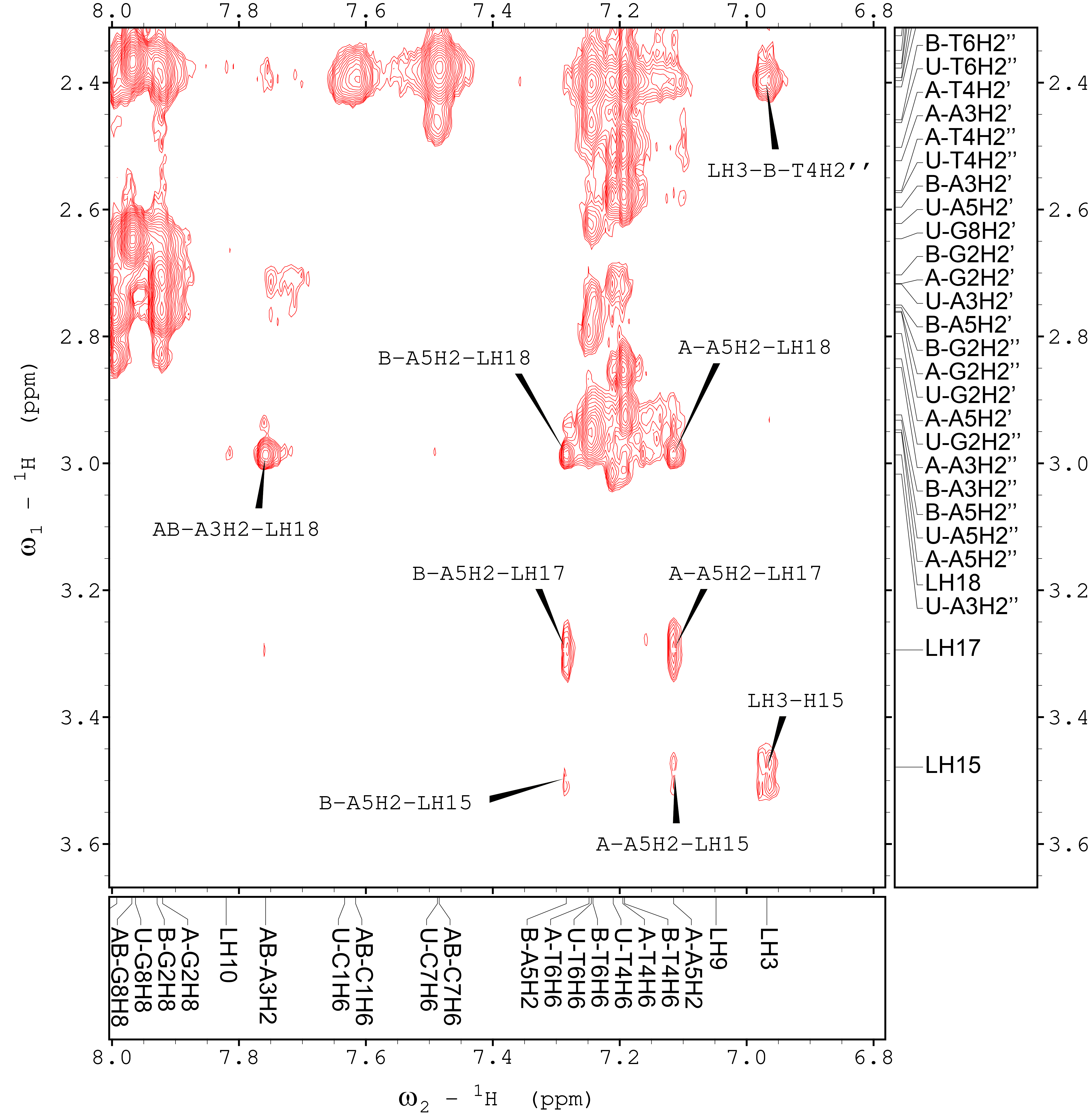


**Figure S11.** Fragment of the NOESY spectrum of the d(CGATATCG)_2_:C-1305 complex, displaying correlations between the aliphatic protons of the ligand and the A3H2/A5H2 protons of the DNA. These NOEs have evidenced that the aminoaliphatic sidechain of C-1305 has settled within the minor groove of the **D2** duplex. Spectrum was recorded in D_2_O.


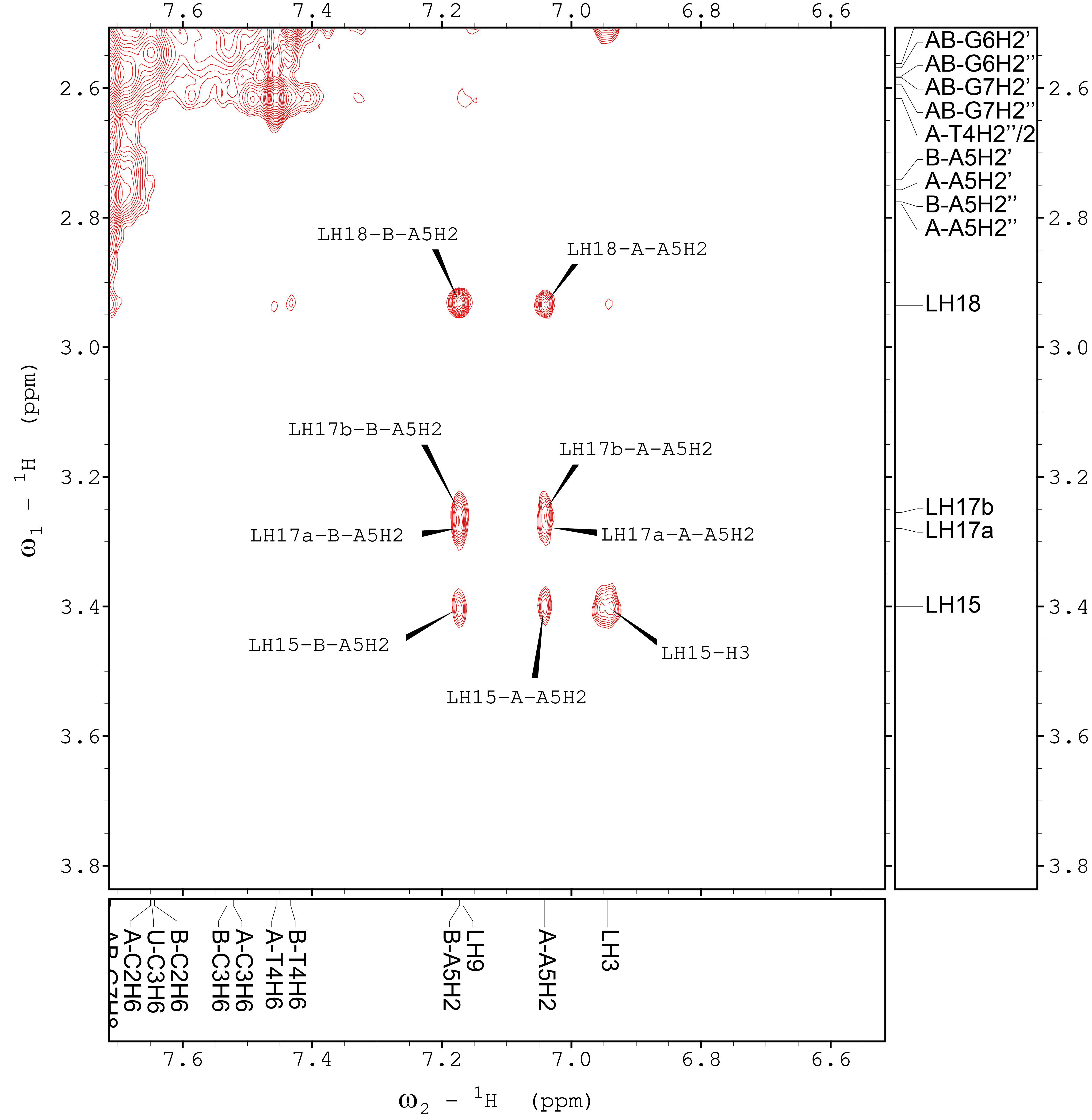


**Figure S12.** Fragment of the NOESY spectrum of the d(CCCTAGGG)_2_:C-1305 complex, displaying correlations between the aliphatic protons of the ligand and the A5H2 protons of the DNA. These NOEs have evidenced that the aminoaliphatic sidechain of C-1305 has settled within the minor groove of the **D3** duplex. Spectrum was recorded in D_2_O.


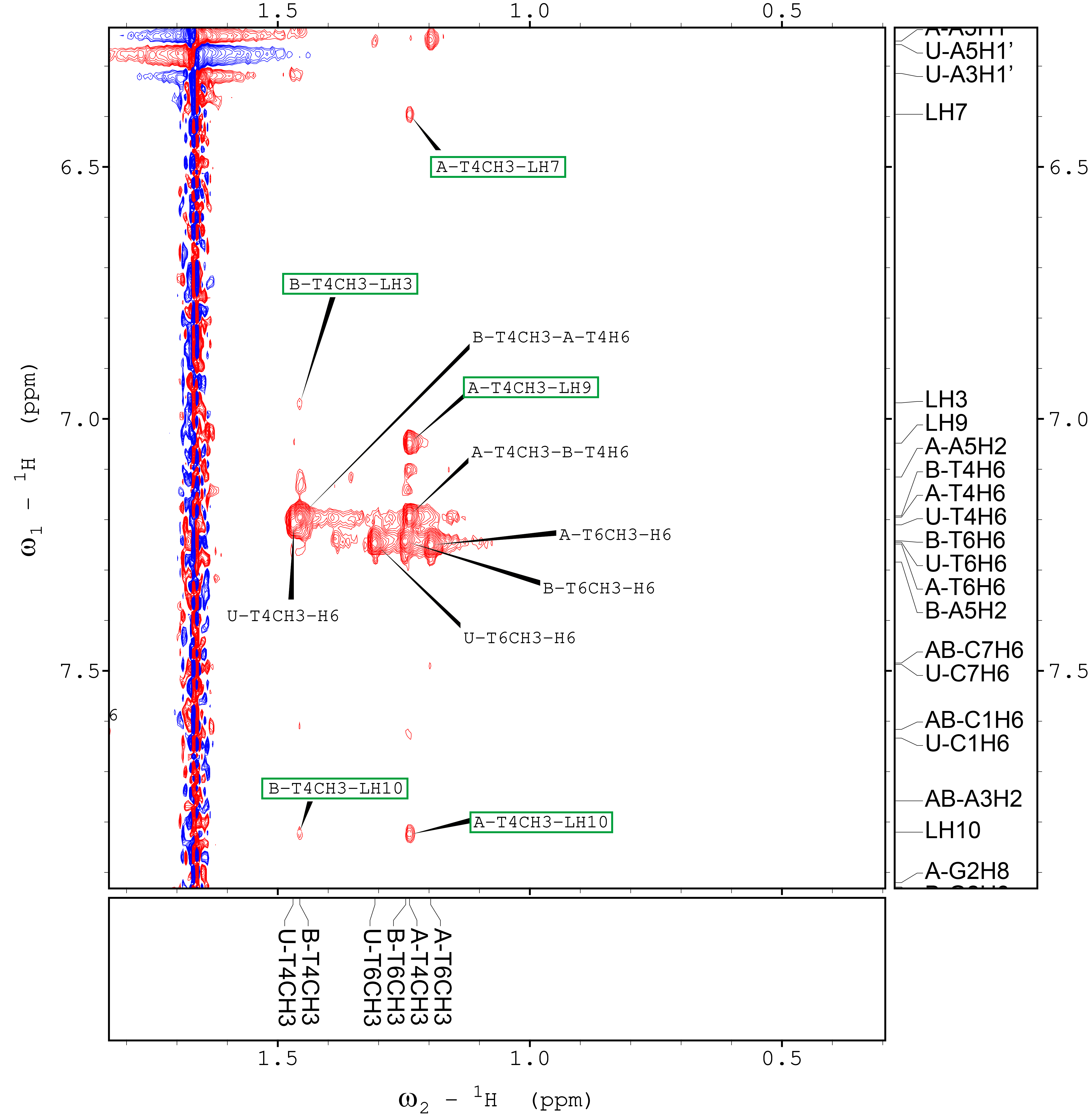


**Figure S13.** Fragment of the NOESY spectrum of the d(CGATATCG)_2_:C-1305 complex, displaying correlations between the aromatic protons of the ligand and the T4CH_3_ protons of the DNA. NOEs involving protons of the ligand have been highlighted by green rectangles. Spectrum was recorded in D_2_O.


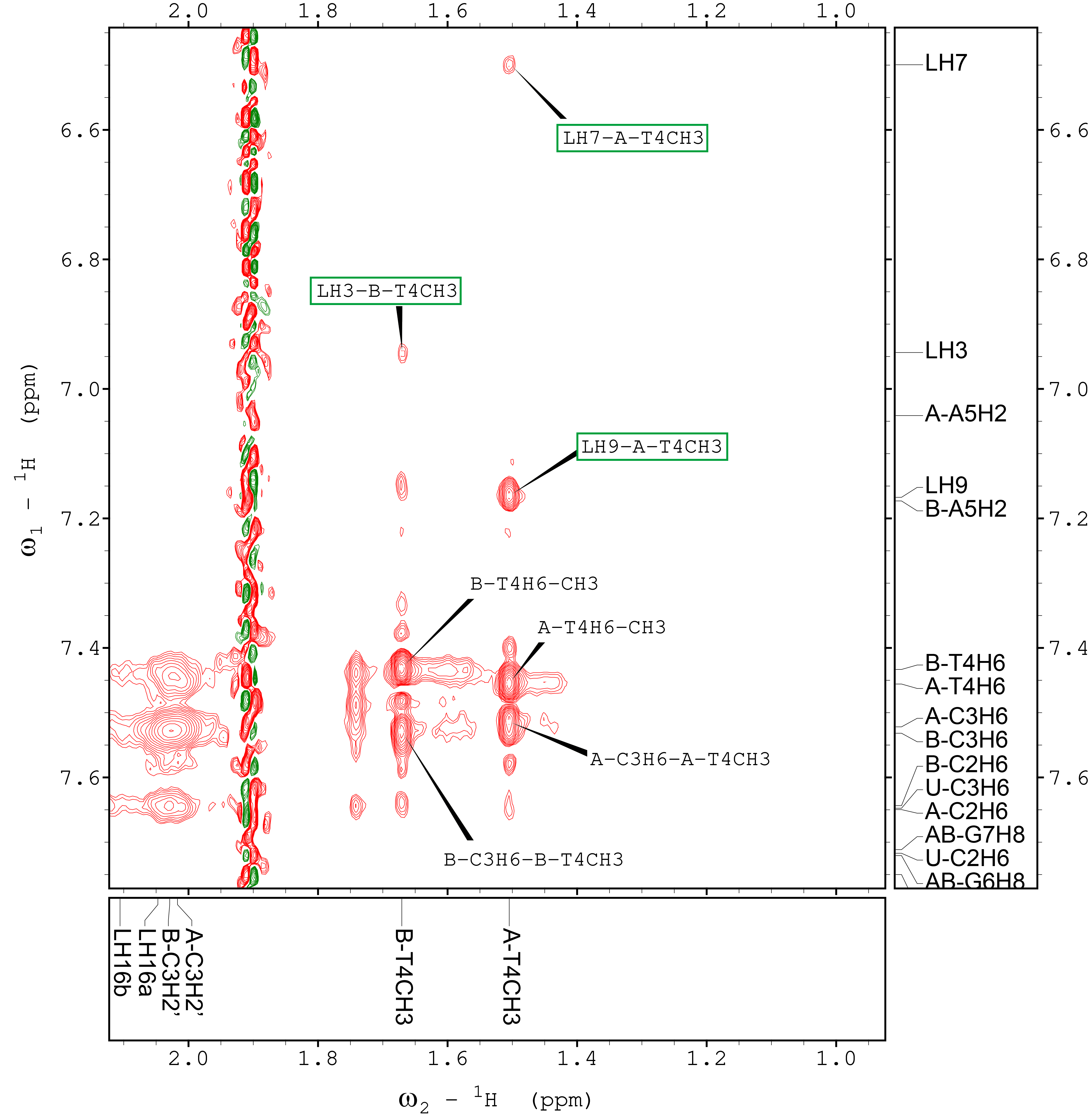


**Figure S14.** Fragment of the NOESY spectrum of the d(CCCTAGGG)_2_:C-1305 complex, displaying correlations between the aromatic protons of the ligand and the T4CH_3_ protons of the DNA. NOEs involving protons of the ligand have been highlighted by green rectangles. Spectrum was recorded in D_2_O.

**Table S1.** Chemical shifts (δ, ppm) of the aromatic and the aliphatic (CH_3_, H1’, H2’, H2’’ and H3’) protons of the d(CGATATCG)_2_:C-1305 complex, along with the changes of δ (Δδ) occurring upon the complex formation (in relation to the free d(CGATATCG)_2_ duplex).

| **Proton** | **δ in strand A [ppm]** | **Δδ in strand A [ppm]** | **δ in strand B [ppm]** | **Δδ in strand B [ppm]** |
| --- | --- | --- | --- | --- |
| C1H1' | 5.682 | -0.011 | 5.672 | -0.021 |
| C1H2' | 1.933 | 0.017 | 1.933 | 0.017 |
| C1H2'' | 2.392 | 0.043 | 2.392 | 0.043 |
| C1H3' | 4.705 | -0.003 | 4.701 | -0.007 |
| C1H5 | 5.848 | -0.037 | 5.848 | -0.037 |
| C1H6 | 7.616 | -0.017 | 7.616 | -0.017 |
| G2H1' | 5.627 | 0.044 | 5.527 | -0.056 |
| G2H2' | 2.717 | -0.045 | 2.703 | -0.059 |
| G2H2'' | 2.760 | -0.075 | 2.755 | -0.081 |
| G2H3' | 5.006 | -0.013 | 5.004 | -0.015 |
| G2H8 | 7.920 | -0.072 | 7.929 | -0.063 |
| A3H1' | 6.198 | -0.117 | 6.218 | -0.097 |
| A3H2 | 7.758 | -0.056 | 7.757 | -0.056 |
| A3H2' | 2.523 | -0.195 | 2.596 | -0.121 |
| A3H2'' | 2.849 | -0.168 | 2.924 | -0.093 |
| A3H3' | 4.972 | -0.089 | 5.057 | -0.004 |
| A3H8 | 8.121 | -0.191 | 8.224 | -0.088 |
| T4CH_3_ | 1.239 | -0.231 | 1.456 | -0.013 |
| T4H1' | 6.023 | 0.281 | 5.855 | 0.113 |
| T4H2' | 2.502 | 0.329 | 2.326 | 0.153 |
| T4H2'' | 2.570 | -0.003 | 2.397 | -0.177 |
| T4H3' | 4.995 | 0.077 | 5.009 | 0.091 |
| T4H6 | 7.195 | -0.015 | 7.192 | -0.018 |
| A5H1' | 6.250 | -0.007 | 6.156 | -0.101 |
| A5H2 | 7.116 | -0.046 | 7.285 | 0.122 |
| A5H2' | 2.796 | 0.174 | 2.751 | 0.129 |
| A5H2'' | 2.952 | 0.004 | 2.932 | -0.015 |
| A5H3' | 5.003 | -0.013 | 4.969 | -0.048 |
| A5H8 | 8.417 | 0.135 | 8.454 | 0.172 |
| T6CH_3_ | 1.197 | -0.273 | 1.246 | -0.224 |
| T6H1' | 5.908 | 0.166 | 5.893 | 0.151 |
| T6H2' | 2.082 | -0.091 | 2.098 | -0.075 |
| T6H2'' | 2.407 | -0.167 | 2.459 | -0.115 |
| T6H3' | 4.857 | -0.062 | 4.854 | -0.064 |
| T6H6 | 7.248 | 0.038 | 7.242 | 0.032 |
| C7H1' | 5.697 | 0.037 | 5.697 | 0.037 |
| C7H2' | 1.972 | 0.006 | 1.972 | 0.006 |
| C7H2'' | 2.377 | 0.006 | 2.377 | 0.006 |
| C7H3' | 4.840 | 0.010 | 4.840 | 0.010 |
| C7H5 | 5.622 | -0.002 | 5.622 | -0.002 |
| C7H6 | 7.485 | -0.003 | 7.485 | -0.003 |
| G8H1' | 6.153 | 0.004 | 6.153 | 0.004 |
| G8H2' | 2.629 | 0.001 | 2.629 | 0.001 |
| G8H2'' | 2.383 | -0.002 | 2.383 | -0.002 |
| G8H3' | 4.699 | 0.000 | 4.699 | 0.000 |
| G8H8 | 7.969 | 0.006 | 7.969 | 0.006 |

**Table S2.** Chemical shifts (δ, ppm) of the aromatic and the aliphatic (CH_3_, H1’, H2’, H2’’ and H3’) protons of the d(CCCTAGGG)_2_:C-1305 complex, along with the changes of δ (Δδ) occurring upon the complex formation (in relation to the free d(CCCTAGGG)_2_ duplex).

| **Proton** | **δ in strand A [ppm]** | **Δδ in strand A [ppm]** | **δ in strand B [ppm]** | **Δδ in strand B [ppm]** |
| --- | --- | --- | --- | --- |
| C1H1' | 5.951 | -0.063 | 5.951 | -0.063 |
| C1H2' | 2.241 | -0.020 | 2.241 | -0.020 |
| C1H2'' | 2.552 | -0.053 | 2.552 | -0.053 |
| C1H3' | 4.659 | -0.044 | 4.659 | -0.044 |
| C1H5 | 5.920 | -0.091 | 5.920 | -0.091 |
| C1H6 | 7.844 | -0.059 | 7.844 | -0.059 |
| C2H1' | 5.962 | -0.105 | 5.990 | -0.077 |
| C2H2' | 2.292 | -0.048 | 2.232 | -0.108 |
| C2H2'' | 2.476 | -0.075 | 2.479 | -0.072 |
| C2H3' | 4.837 | -0.070 | 4.856 | -0.051 |
| C2H5 | 5.580 | -0.128 | 5.580 | -0.128 |
| C2H6 | 7.648 | -0.087 | 7.644 | -0.091 |
| C3H1' | 5.837 | -0.157 | 5.880 | -0.114 |
| C3H2' | 2.023 | -0.156 | 2.031 | -0.148 |
| C3H2'' | 2.438 | -0.093 | 2.458 | -0.073 |
| C3H3' | 4.716 | -0.136 | 4.801 | -0.051 |
| C3H5 | 5.507 | -0.187 | 5.521 | -0.173 |
| C3H6 | 7.521 | -0.141 | 7.532 | -0.130 |
| T4H1' | 6.137 | 0.634 | 5.938 | 0.435 |
| T4H2' | 2.617 | 0.433 | 2.493 | 0.309 |
| T4H2'' | ---^a^ | --- | ---^a^ | --- |
| T4H3' | 5.019 | 0.133 | 5.015 | 0.129 |
| T4H6 | 7.455 | -0.046 | 7.433 | -0.068 |
| A5H1' | 5.835 | -0.164 | 5.747 | -0.252 |
| A5H2 | 7.039 | -0.443 | 7.172 | -0.310 |
| A5H2' | 2.789 | 0.050 | 2.739 | 0.000 |
| A5H2'' | 2.771 | -0.098 | 2.763 | -0.106 |
| A5H3' | 4.992 | -0.068 | 4.946 | -0.114 |
| A5H8 | 8.289 | 0.046 | 8.331 | 0.088 |
| G6H1' | 5.377 | 0.000 | 5.377 | 0.000 |
| G6H2' | 2.561 | -0.001 | 2.561 | -0.001 |
| G6H2'' | 2.579 | -0.030 | 2.579 | -0.030 |
| G6H3' | 4.955 | -0.005 | 4.955 | -0.005 |
| G6H8 | 7.715 | -0.034 | 7.720 | -0.029 |
| G7H1' | 5.822 | 0.015 | 5.843 | 0.036 |
| G7H2' | 2.587 | 0.016 | 2.580 | 0.009 |
| G7H2'' | 2.761 | 0.018 | 2.771 | 0.028 |
| G7H3' | 4.986 | 0.034 | 4.986 | 0.034 |
| G7H8 | 7.710 | 0.016 | 7.710 | 0.016 |
| G8H1' | 6.121 | -0.024 | 6.121 | -0.024 |
| G8H2' | 2.507 | 0.009 | 2.507 | 0.009 |
| G8H2'' | 2.328 | -0.009 | 2.328 | -0.009 |
| G8H3' | 4.650 | 0.004 | 4.650 | 0.004 |
| G8H8 | 7.748 | -0.014 | 7.748 | -0.014 |

^a^ Resonance not assigned - possibly degenerate with T4H2’ of the same strand.

**Table S3.** Chemical shifts (δ, ppm) of the phosphorus atoms of the d(CCCTAGGG)_2_:C-1305 complex, along with the changes of δ (Δδ) occurring upon the complex formation (in relation to the free d(CCCTAGGG)_2_ duplex).

| **Atom** | **δ in strand A [ppm]** | **Δδ in strand A [ppm]** | **δ in strand B [ppm]** | **Δδ in strand B [ppm]** |
| --- | --- | --- | --- | --- |
| C2P | -4.106 | -0.025 | -4.106 | -0.025 |
| C3P | -3.874 | -0.013 | -3.919 | -0.058 |
| T4P | -4.171 | 0.073 | -4.050 | 0.194 |
| A5P | -3.423 | 0.494 | -3.055 | 0.862 |
| G6P | ---^a^ | --- | -3.992 | 0.084 |
| G7P | -3.630 | -0.030 | -3.564 | 0.036 |
| G8P | -4.035 | -0.038 | -4.035 | -0.038 |

^a^ Resonance not assigned.

**Table S4.** Distance restrains file, used in restrained **D2L-MD1** calculations (GROMACS .itp format). The following 13 distance restraints correspond to the 13 observed DNA/ligand intermolecular NOEs, involving the aromatic protons of C-1305. *Low* and *up1* columns define the minimum and the maximum proton-proton distances with no penalty potential applied, respectively. *Up2* stands for the proton-proton distance above which a wall of the potential is applied. All NOEs classified as weak, medium and strong were given the same boundaries, respectively.

| [ distance_restraints ] | | |  |  |  |  |  |  |  |
| --- | --- | --- | --- | --- | --- | --- | --- | --- | --- |
| ; | ai | aj | type | index | type' | low | up1 | up2 | fac |
| ; DNA-ligand interactions | | |  |  |  |  |  |  |  |
| ; | C1305H3/bA5H8; topology C0517:H6/DA5:H8; medium | | | | | |  |  |  |
|  | 530 | 142 | 1 | 14 | 1 | 0.2500 | 0.4500 | 0.5500 | 1 |
| ; | C1305H3/bT4H1'; topology C0517:H6/DT4:H1'; weak | | | | | |  |  |  |
|  | 530 | 105 | 1 | 15 | 1 | 0.3000 | 0.6000 | 0.7000 | 1 |
| ; | C1305H3/bT4H2''; topology C0517:H6/DT4:H2''; strong | | | | | |  |  |  |
|  | 530 | 122 | 1 | 16 | 1 | 0.2000 | 0.3250 | 0.4250 | 1 |
| ; | C1305H3/bT4H6; topology C0517:H6/DT4H6; weak | | | | |  |  |  |  |
|  | 530 | 108 | 1 | 17 | 1 | 0.3000 | 0.6000 | 0.7000 | 1 |
| ; | C1305H7/aT4H1'; topology C0517:H1/DT12:H1'; medium | | | | | |  |  |  |
|  | 513 | 358 | 1 | 18 | 1 | 0.2500 | 0.4500 | 0.5500 | 1 |
| ; C1305H7/aT4H2'; topology C0517:H1/DT12:H2'; strong | | | | | |  |  |  |  |
|  | 513 | 374 | 1 | 19 | 1 | 0.2000 | 0.3250 | 0.4250 | 1 |
| ; C1305H7/aT4H2''; topology C0517:H1/DT12:H2''; strong | | | | | |  |  |  |  |
|  | 513 | 375 | 1 | 20 | 1 | 0.2000 | 0.3250 | 0.4250 | 1 |
| ; C1305H7/aT4H3'; topology C0517:H1/DT12:H3'; weak | | | | | |  |  |  |  |
|  | 513 | 377 | 1 | 21 | 1 | 0.3000 | 0.6000 | 0.7000 | 1 |
| ; C1305H7/aT4CH3; topology C0517:H1/DT12:H52; medium | | | | | |  |  |  |  |
|  | 513 | 370 | 1 | 22 | 1 | 0.2500 | 0.4500 | 0.5500 | 1 |
|  | 513 | 371 | 1 | 22 | 1 | 0.2500 | 0.4500 | 0.5500 | 1 |
|  | 513 | 372 | 1 | 22 | 1 | 0.2500 | 0.4500 | 0.5500 | 1 |
| ; C1305H7/aT4H6; topology C0517:H1/DT12:H6; weak | | | | | |  |  |  |  |
|  | 513 | 361 | 1 | 23 | 1 | 0.3000 | 0.6000 | 0.7000 | 1 |
| ; C1305H7/aA5H8; topology C0517:H1/DA13:H8; weak | | | | | |  |  |  |  |
|  | 513 | 395 | 1 | 24 | 1 | 0.3000 | 0.6000 | 0.7000 | 1 |
| ; C1305H9/aT4CH3; topology C0517:H3/DT12:H52; strong | | | | | |  |  |  |  |
|  | 518 | 370 | 1 | 25 | 1 | 0.2000 | 0.3250 | 0.4250 | 1 |
|  | 518 | 371 | 1 | 25 | 1 | 0.2000 | 0.3250 | 0.4250 | 1 |
|  | 518 | 372 | 1 | 25 | 1 | 0.2000 | 0.3250 | 0.4250 | 1 |
| ; C1305H10/aT4CH3; topology C0517:H2/DT12:H52; weak | | | | | |  |  |  |  |
|  | 515 | 370 | 1 | 26 | 1 | 0.3000 | 0.6000 | 0.7000 | 1 |
|  | 515 | 371 | 1 | 26 | 1 | 0.3000 | 0.6000 | 0.7000 | 1 |
|  | 515 | 372 | 1 | 26 | 1 | 0.3000 | 0.6000 | 0.7000 | 1 |

**Table S5.** Distance restrains file, used in restrained **D3L-MD1** calculations (GROMACS .itp format). The following 18 distance restraints correspond to the 19 observed DNA/ligand intermolecular NOEs, involving the aromatic protons of C-1305. *Low*, *up1* and *up2* values defined as in **Table S4**.

| [ distance_restraints ] | |  |  |  |  |  |  |  |  |
| --- | --- | --- | --- | --- | --- | --- | --- | --- | --- |
| ; | ai | aj | type | index | type' | low | up1 | up2 | fac |
| ; | LH3/BT4H2'' |  | strong |  |  |  |  |  |  |
|  | 117 | 528 | 1 | 18 | 1 | 0.2000 | 0.3250 | 0.4250 | 1 |
| ; | LH3/BT4H6 |  | weak |  |  |  |  |  |  |
|  | 528 | 103 | 1 | 20 | 1 | 0.3000 | 0.6000 | 0.7000 | 1 |
| ; | LH3/BA5H8 |  | weak |  |  |  |  |  |  |
|  | 528 | 137 | 1 | 21 | 1 | 0.3000 | 0.6000 | 0.7000 | 1 |
| ; | LH4/AA5H2 |  | weak |  |  |  |  |  |  |
|  | 526 | 392 | 1 | 22 | 1 | 0.3000 | 0.6000 | 0.7000 | 1 |
| ; | LH4/BA5H8 |  | weak |  |  |  |  |  |  |
|  | 526 | 137 | 1 | 23 | 1 | 0.3000 | 0.6000 | 0.7000 | 1 |
| ; | LH7/AT4H6 |  | weak |  |  |  |  |  |  |
|  | 511 | 355 | 1 | 24 | 1 | 0.3000 | 0.6000 | 0.7000 | 1 |
| ; | LH7/AA5H8 |  | weak |  |  |  |  |  |  |
|  | 511 | 389 | 1 | 25 | 1 | 0.3000 | 0.6000 | 0.7000 | 1 |
| ; | LH10/AT4CH3 | | weak |  |  |  |  |  |  |
|  | 364 | 513 | 1 | 26 | 1 | 0.3000 | 0.6000 | 0.7000 | 1 |
|  | 365 | 513 | 1 | 26 | 1 | 0.3000 | 0.6000 | 0.7000 | 1 |
|  | 366 | 513 | 1 | 26 | 1 | 0.3000 | 0.6000 | 0.7000 | 1 |
| ; | LH7/AT4CH3 | | weak |  |  |  |  |  |  |
|  | 364 | 511 | 1 | 27 | 1 | 0.3000 | 0.6000 | 0.7000 | 1 |
|  | 365 | 511 | 1 | 27 | 1 | 0.3000 | 0.6000 | 0.7000 | 1 |
|  | 366 | 511 | 1 | 27 | 1 | 0.3000 | 0.6000 | 0.7000 | 1 |
| ; | LH9/AT4CH3 | | strong |  |  |  |  |  |  |
|  | 364 | 516 | 1 | 28 | 1 | 0.2000 | 0.3250 | 0.4250 | 1 |
|  | 365 | 516 | 1 | 28 | 1 | 0.2000 | 0.3250 | 0.4250 | 1 |
|  | 366 | 516 | 1 | 28 | 1 | 0.2000 | 0.3250 | 0.4250 | 1 |
| ; | LH3/BT4H1' |  | medium |  |  |  |  |  |  |
|  | 100 | 528 | 1 | 29 | 1 | 0.2500 | 0.4500 | 0.5500 | 1 |
| ; | LH3/BT4H3' |  | weak |  |  |  |  |  |  |
|  | 119 | 528 | 1 | 30 | 1 | 0.3000 | 0.6000 | 0.7000 | 1 |
| ; | LH3/BT4CH3 | | weak |  |  |  |  |  |  |
|  | 112 | 528 | 1 | 32 | 1 | 0.3000 | 0.6000 | 0.7000 | 1 |
|  | 113 | 528 | 1 | 32 | 1 | 0.3000 | 0.6000 | 0.7000 | 1 |
|  | 114 | 528 | 1 | 32 | 1 | 0.3000 | 0.6000 | 0.7000 | 1 |
| ; | LH7/AA5H1' |  | weak |  |  |  |  |  |  |
|  | 384 | 511 | 1 | 33 | 1 | 0.3000 | 0.6000 | 0.7000 | 1 |
| ; | LH4/BA5H4' |  | weak |  |  |  |  |  |  |
|  | 129 | 526 | 1 | 34 | 1 | 0.3000 | 0.6000 | 0.7000 | 1 |
| ; | LH3/BA5H1' |  | weak |  |  |  |  |  |  |
|  | 132 | 528 | 1 | 36 | 1 | 0.3000 | 0.6000 | 0.7000 | 1 |
| ; | LH7/AT4H2'' | or | LH7/AT4H2' | | medium |  |  |  |  |
|  | 368 | 511 | 1 | 37 | 1 | 0.2000 | 0.3250 | 0.4250 | 1 |
|  | 369 | 511 | 1 | 37 | 1 | 0.2000 | 0.3250 | 0.4250 | 1 |
| ; | LH7/AT4H1' |  | medium |  |  |  |  |  |  |
|  | 352 | 511 | 1 | 38 | 1 | 0.2500 | 0.4500 | 0.5500 | 1 |

**Table S6.** Results of initial 700 ns of **D2L** preliminary MD simulations, considering observed NOEs. Green values stand for met NOE proton-proton distance criteria during MD calculations, orange values depict a poor match, red values stand for average distances at which respective NOEs should not be observed.

| **D2L – NO DISTANCE RESTRAINTS,**  **MAIN STRUCTURAL CLUSTER** | | |  | **D2L – ALL RESTRAINTS DERIVED FROM NOEs, MAIN STRUCTURAL CLUSTER** | | |
| --- | --- | --- | --- | --- | --- | --- |
| **NOE** | **AVERAGE DISTANCE [nm]** | **STANDARD DEVIATION [nm]** |  | **NOE** | **AVERAGE DISTANCE [nm]** | **STANDARD DEVIATION [nm]** |
| **LH3/BA5H8** | 0.446 | 0.081 |  | **LH3/BA5H8** | 0.390 | 0.044 |
| **LH3/BT4H1'** | 0.386 | 0.069 |  | **LH3/BT4H1'** | 0.476 | 0.041 |
| **LH3/BT4H2''** | 0.328 | 0.078 |  | **LH3/BT4H2''** | 0.327 | 0.035 |
| **LH3/HT4H6** | 0.396 | 0.056 |  | **LH3/HT4H6** | 0.387 | 0.043 |
| **LH7/AT4H1'** | 0.449 | 0.065 |  | **LH7/AT4H1'** | 0.368 | 0.047 |
| **LH7/AT4H2'** | 0.270 | 0.073 |  | **LH7/AT4H2'** | 0.352 | 0.044 |
| **LH7/AT4H2''** | 0.383 | 0.057 |  | **LH7/AT4H2''** | 0.275 | 0.035 |
| **LH7/AT4H3'** | 0.429 | 0.075 |  | **LH7/AT4H3'** | 0.528 | 0.035 |
| **LH7/AT4CH3** | 0.559 | 0.079 |  | **LH7/AT4CH3** | 0.575 | 0.059 |
| **LH7/AT4CH3** | 0.560 | 0.082 |  | **LH7/AT4CH3** | 0.576 | 0.059 |
| **LH7/AT4CH3** | 0.564 | 0.080 |  | **LH7/AT4CH3** | 0.575 | 0.062 |
| **LH7/AT4H6** | 0.426 | 0.056 |  | **LH7/AT4H6** | 0.425 | 0.036 |
| **LH7/AA5H8** | 0.489 | 0.041 |  | **LH7/AA5H8** | 0.522 | 0.061 |
| **LH9/AT4CH3** | 0.398 | 0.089 |  | **LH9/AT4CH3** | 0.375 | 0.080 |
| **LH9/AT4CH3** | 0.400 | 0.089 |  | **LH9/AT4CH3** | 0.377 | 0.075 |
| **LH9/AT4CH3** | 0.405 | 0.090 |  | **LH9/AT4CH3** | 0.375 | 0.079 |
| **LH10/AT4CH3** | 0.455 | 0.106 |  | **LH10/AT4CH3** | 0.427 | 0.085 |
| **LH10/AT4CH3** | 0.452 | 0.106 |  | **LH10/AT4CH3** | 0.428 | 0.083 |
| **LH10/AT4CH3** | 0.461 | 0.104 |  | **LH10/AT4CH3** | 0.428 | 0.082 |
| **LH10/BT4CH3** | 0.702 | 0.131 |  | **LH10/BT4CH3** | 0.541 | 0.091 |
| **LH10/BT4CH3** | 0.693 | 0.130 |  | **LH10/BT4CH3** | 0.537 | 0.096 |
| **LH10/BT4CH3** | 0.696 | 0.135 |  | **LH10/BT4CH3** | 0.548 | 0.093 |
| **LH15/AA5H2** | 0.543 | 0.056 |  | **LH15/AA5H2** | 0.427 | 0.050 |
| **LH15/AA5H2** | 0.540 | 0.064 |  | **LH15/AA5H2** | 0.284 | 0.058 |
| **LH15/BA5H2** | 0.522 | 0.096 |  | **LH15/BA5H2** | 0.502 | 0.075 |
| **LH15/BA5H2** | 0.512 | 0.087 |  | **LH15/BA5H2** | 0.536 | 0.040 |
| **LH17/AA5H2** | 0.493 | 0.108 |  | **LH17/AA5H2** | 0.401 | 0.070 |
| **LH17/AA5H2** | 0.518 | 0.083 |  | **LH17/AA5H2** | 0.490 | 0.099 |
| **LH17/BA5H2** | 0.741 | 0.098 |  | **LH17/BA5H2** | 0.329 | 0.104 |
| **LH17/BA5H2** | 0.746 | 0.093 |  | **LH17/BA5H2** | 0.332 | 0.068 |
| **LH18/AA5H2** | 0.529 | 0.109 |  | **LH18/AA5H2** | 0.536 | 0.078 |
| **LH18/AA5H2** | 0.498 | 0.120 |  | **LH18/AA5H2** | 0.525 | 0.072 |
| **LH18/AA5H2** | 0.509 | 0.119 |  | **LH18/AA5H2** | 0.521 | 0.074 |
| **LH18/AA5H2** | 0.574 | 0.143 |  | **LH18/AA5H2** | 0.439 | 0.086 |
| **LH18/AA5H2** | 0.566 | 0.139 |  | **LH18/AA5H2** | 0.468 | 0.092 |
| **LH18/AA5H2** | 0.574 | 0.131 |  | **LH18/AA5H2** | 0.447 | 0.083 |
| **LH18/BA5H2** | 0.956 | 0.107 |  | **LH18/BA5H2** | 0.501 | 0.060 |
| **LH18/BA5H2** | 0.930 | 0.117 |  | **LH18/BA5H2** | 0.588 | 0.061 |
| **LH18/BA5H2** | 0.918 | 0.100 |  | **LH18/BA5H2** | 0.509 | 0.089 |
| **LH18/BA5H2** | 0.960 | 0.112 |  | **LH18/BA5H2** | 0.497 | 0.089 |
| **LH18/BA5H2** | 0.943 | 0.117 |  | **LH18/BA5H2** | 0.520 | 0.088 |
| **LH18/BA5H2** | 0.988 | 0.114 |  | **LH18/BA5H2** | 0.484 | 0.098 |
| **LH18/AA3H2** | 1.309 | 0.141 |  | **LH18/AA3H2** | 0.538 | 0.103 |
| **LH18/AA3H2** | 1.283 | 0.147 |  | **LH18/AA3H2** | 0.620 | 0.092 |
| **LH18/AA3H2** | 1.271 | 0.126 |  | **LH18/AA3H2** | 0.593 | 0.107 |
| **LH18/AA3H2** | 1.307 | 0.135 |  | **LH18/AA3H2** | 0.587 | 0.094 |
| **LH18/AA3H2** | 1.286 | 0.136 |  | **LH18/AA3H2** | 0.579 | 0.099 |
| **LH18/AA3H2** | 1.333 | 0.145 |  | **LH18/AA3H2** | 0.563 | 0.126 |
| **LH18/BA3H2** | 0.532 | 0.167 |  | **LH18/BA3H2** | 0.751 | 0.091 |
| **LH18/BA3H2** | 0.519 | 0.165 |  | **LH18/BA3H2** | 0.709 | 0.091 |
| **LH18/BA3H2** | 0.544 | 0.160 |  | **LH18/BA3H2** | 0.727 | 0.099 |
| **LH18/BA3H2** | 0.605 | 0.160 |  | **LH18/BA3H2** | 0.666 | 0.077 |
| **LH18/BA3H2** | 0.610 | 0.162 |  | **LH18/BA3H2** | 0.683 | 0.087 |
| **LH18/BA3H2** | 0.583 | 0.163 |  | **LH18/BA3H2** | 0.675 | 0.092 |

**Table S7.** Results of initial 700 ns of **D3L** preliminary MD simulations, considering observed NOEs. Green values stand for met NOE proton-proton distance criteria during MD calculations, orange values depict a poor match, red values stand for average distances at which respective NOEs should not be observed.

| **D3L – NO DISTANCE RESTRAINTS,**  **MAIN STRUCTURAL CLUSTER** | | |  | **D3L – ALL RESTRAINTS DERIVED FROM NOEs, MAIN STRUCTURAL CLUSTER** | | |
| --- | --- | --- | --- | --- | --- | --- |
| **NOE** | **AVERAGE DISTANCE [nm]** | **STANDARD DEVIATION [nm]** |  | **NOE** | **AVERAGE DISTANCE [nm]** | **STANDARD DEVIATION [nm]** |
| **LH3/BT4H2''** | 0.374 | 0.108 |  | **LH3/BT4H2''** | 0.287 | 0.047 |
| **LH7/AT4H2''** | 0.276 | 0.077 |  | **LH7/AT4H2''** | 0.328 | 0.051 |
| **LH3/BT4H6** | 0.429 | 0.071 |  | **LH3/BT4H6** | 0.472 | 0.046 |
| **LH3/BA5H8** | 0.453 | 0.080 |  | **LH3/BA5H8** | 0.438 | 0.056 |
| **LH4/AA5H2** | 0.506 | 0.057 |  | **LH4/AA5H2** | 0.433 | 0.067 |
| **LH4/BA5H8** | 0.575 | 0.138 |  | **LH4/BA5H8** | 0.510 | 0.047 |
| **LH7/AT4H6** | 0.448 | 0.052 |  | **LH7/AT4H6** | 0.372 | 0.041 |
| **LH7/AA5H8** | 0.476 | 0.050 |  | **LH7/AA5H8** | 0.411 | 0.046 |
| **LH10/AT4CH3** | 0.505 | 0.119 |  | **LH10/AT4CH3** | 0.425 | 0.084 |
| **LH10/AT4CH3** | 0.496 | 0.114 |  | **LH10/AT4CH3** | 0.431 | 0.083 |
| **LH10/AT4CH3** | 0.503 | 0.120 |  | **LH10/AT4CH3** | 0.431 | 0.083 |
| **LH7/AT4CH3** | 0.570 | 0.087 |  | **LH7/AT4CH3** | 0.475 | 0.085 |
| **LH7/AT4CH3** | 0.568 | 0.087 |  | **LH7/AT4CH3** | 0.479 | 0.087 |
| **LH7/AT4CH3** | 0.575 | 0.083 |  | **LH7/AT4CH3** | 0.474 | 0.086 |
| **LH9/AT4CH3** | 0.410 | 0.097 |  | **LH9/AT4CH3** | 0.434 | 0.069 |
| **LH9/AT4CH3** | 0.401 | 0.094 |  | **LH9/AT4CH3** | 0.439 | 0.073 |
| **LH9/AT4CH3** | 0.412 | 0.093 |  | **LH9/AT4CH3** | 0.436 | 0.073 |
| **LH3/BT4H1'** | 0.432 | 0.088 |  | **LH3/BT4H1'** | 0.325 | 0.046 |
| **LH3/BT4H3'** | 0.477 | 0.064 |  | **LH3/BT4H3'** | 0.546 | 0.044 |
| **LH10/BT4CH3** | 0.712 | 0.144 |  | **LH10/BT4CH3** | 0.571 | 0.101 |
| **LH10/BT4CH3** | 0.710 | 0.144 |  | **LH10/BT4CH3** | 0.572 | 0.108 |
| **LH10/BT4CH3** | 0.709 | 0.145 |  | **LH10/BT4CH3** | 0.563 | 0.107 |
| **LH3/BT4CH3** | 0.544 | 0.128 |  | **LH3/BT4CH3** | 0.643 | 0.067 |
| **LH3/BT4CH3** | 0.542 | 0.124 |  | **LH3/BT4CH3** | 0.637 | 0.066 |
| **LH3/BT4CH3** | 0.544 | 0.126 |  | **LH3/BT4CH3** | 0.638 | 0.068 |
| **LH7/AA5H1'** | 0.473 | 0.082 |  | **LH7/AA5H1'** | 0.610 | 0.064 |
| **LH4/AA5H4'** | 0.941 | 0.084 |  | **LH4/AA5H4'** | 0.650 | 0.066 |
| **LH4/AA5H5'** | 0.939 | 0.082 |  | **LH4/AA5H5'** | 0.649 | 0.067 |
| **LH3/BA5H1'** | 0.506 | 0.093 |  | **LH3/BA5H1'** | 0.555 | 0.061 |
| **LH15/BA5H4'** | 0.549 | 0.122 |  | **LH15/BA5H4'** | 0.673 | 0.060 |
| **LH15/BA5H4'** | 0.552 | 0.162 |  | **LH15/BA5H4'** | 0.557 | 0.049 |
| **LH17/BA5H4'** | 0.651 | 0.170 |  | **LH17/BA5H4'** | 0.503 | 0.084 |
| **LH17/BA5H4'** | 0.647 | 0.167 |  | **LH17/BA5H4'** | 0.410 | 0.074 |
| **LH18/BA5H1'** | 0.824 | 0.221 |  | **LH18/BA5H1'** | 0.603 | 0.117 |
| **LH18/BA5H1'** | 0.822 | 0.215 |  | **LH18/BA5H1'** | 0.563 | 0.112 |
| **LH18/BA5H1'** | 0.817 | 0.205 |  | **LH18/BA5H1'** | 0.636 | 0.106 |
| **LH18/BA5H1'** | 0.834 | 0.227 |  | **LH18/BA5H1'** | 0.650 | 0.115 |
| **LH18/BA5H1'** | 0.831 | 0.218 |  | **LH18/BA5H1'** | 0.661 | 0.113 |
| **LH18/BA5H1'** | 0.841 | 0.227 |  | **LH18/BA5H1'** | 0.646 | 0.119 |
| **LH15/BA5H1'** | 0.594 | 0.120 |  | **LH15/BA5H1'** | 0.565 | 0.056 |
| **LH15/BA5H1'** | 0.600 | 0.158 |  | **LH15/BA5H1'** | 0.426 | 0.059 |
| **LH15/AA5H2** | 0.525 | 0.069 |  | **LH15/AA5H2** | 0.539 | 0.056 |
| **LH15/AA5H2** | 0.514 | 0.070 |  | **LH15/AA5H2** | 0.517 | 0.046 |
| **LH15/BA5H2** | 0.511 | 0.067 |  | **LH15/BA5H2** | 0.408 | 0.052 |
| **LH15/BA5H2** | 0.529 | 0.068 |  | **LH15/BA5H2** | 0.326 | 0.058 |
| **LH17/AA5H2** | 0.586 | 0.157 |  | **LH17/AA5H2** | 0.558 | 0.046 |
| **LH17/AA5H2** | 0.591 | 0.147 |  | **LH17/AA5H2** | 0.509 | 0.065 |
| **LH17/BA5H2** | 0.617 | 0.135 |  | **LH17/BA5H2** | 0.573 | 0.048 |
| **LH17/BA5H2** | 0.604 | 0.161 |  | **LH17/BA5H2** | 0.549 | 0.059 |
| **LH18/AA5H2** | 0.705 | 0.227 |  | **LH18/AA5H2** | 0.505 | 0.147 |
| **LH18/AA5H2** | 0.675 | 0.216 |  | **LH18/AA5H2** | 0.555 | 0.124 |
| **LH18/AA5H2** | 0.685 | 0.226 |  | **LH18/AA5H2** | 0.568 | 0.109 |
| **LH18/AA5H2** | 0.735 | 0.201 |  | **LH18/AA5H2** | 0.635 | 0.094 |
| **LH18/AA5H2** | 0.746 | 0.213 |  | **LH18/AA5H2** | 0.633 | 0.095 |
| **LH18/AA5H2** | 0.736 | 0.203 |  | **LH18/AA5H2** | 0.639 | 0.096 |
| **LH18/BA5H2** | 0.732 | 0.234 |  | **LH18/BA5H2** | 0.759 | 0.080 |
| **LH18/BA5H2** | 0.715 | 0.243 |  | **LH18/BA5H2** | 0.735 | 0.073 |
| **LH18/BA5H2** | 0.719 | 0.221 |  | **LH18/BA5H2** | 0.812 | 0.088 |
| **LH18/BA5H2** | 0.767 | 0.226 |  | **LH18/BA5H2** | 0.791 | 0.079 |
| **LH18/BA5H2** | 0.760 | 0.218 |  | **LH18/BA5H2** | 0.806 | 0.086 |
| **LH18/BA5H2** | 0.764 | 0.222 |  | **LH18/BA5H2** | 0.795 | 0.085 |

**Table S8.** Average NOE violations traced for dominant conformational clusters during **D2L-MD1** and **D3L-MD1** simulations. All distances are given in [nm]. ‘DR’ stands for ‘distance restraint’.

| **D2L MAIN STRUCTURAL CLUSTER** | | | | | **D3L MAIN STRUCTURAL CLUSTER** | | | | |
| --- | --- | --- | --- | --- | --- | --- | --- | --- | --- |
| **NOE** | **Average distance observed** | **Standard deviation** | **DR upper bound** | **Average NOE violation** | **NOE** | **Average distance observed** | **Standard deviation** | **DR upper bound** | **Average NOE violation** |
| **LH3/BA5H8** | 0.4487 | 0.0621 | 0.4500 | 0 | **LH3/BT4H2''** | 0.2982 | 0.0554 | 0.3250 | 0 |
| **LH3/BT4H1'** | 0.3628 | 0.0566 | 0.6000 | 0 | **LH3/BT4H6** | 0.3962 | 0.0480 | 0.6000 | 0 |
| **LH3/BT4H2''** | 0.2945 | 0.0524 | 0.3250 | 0 | **LH3/BA5H8** | 0.4094 | 0.0430 | 0.6000 | 0 |
| **LH3/BT4H6** | 0.4282 | 0.0663 | 0.6000 | 0 | **LH4/AA5H2** | 0.5310 | 0.0461 | 0.6000 | 0 |
| **LH7/AT4H1'** | 0.4205 | 0.0484 | 0.4500 | 0 | **LH4/BA5H8** | 0.4932 | 0.0801 | 0.6000 | 0 |
| **LH7/AT4H2'** | 0.2583 | 0.0457 | 0.3250 | 0 | **LH7/AT4H6** | 0.4580 | 0.0476 | 0.6000 | 0 |
| **LH7/BT4H2''** | 0.3257 | 0.0637 | 0.3250 | 0.0007 | **LH7/AA5H8** | 0.4907 | 0.0430 | 0.6000 | 0 |
| **LH7/AT4H3'** | 0.4507 | 0.0599 | 0.6000 | 0 | **LH10/AT4CH3** | 0.4509 | 0.0894 | 0.6000 | 0 |
| **LH7/AT4CH3** | 0.4835 | 0.0505 | 0.4500 | 0.0335 | **LH7/AT4CH3** | 0.5892 | 0.0698 | 0.6000 | 0 |
| **LH7/AT4H6** | 0.4014 | 0.0463 | 0.4500 | 0 | **LH9/AT4CH3** | 0.2921 | 0.0419 | 0.3250 | 0 |
| **LH7/AA5H8** | 0.4737 | 0.0506 | 0.6000 | 0 | **LH3/BT4H1'** | 0.3750 | 0.0574 | 0.4500 | 0 |
| **LH9/AT4CH3** | 0.3097 | 0.0442 | 0.3250 | 0 | **LH3/BT4H3'** | 0.5028 | 0.0512 | 0.6000 | 0 |
| **LH10/AT4CH3** | 0.4857 | 0.1039 | 0.6000 | 0 | **LH3/BT4CH3** | 0.5368 | 0.0884 | 0.6000 | 0 |
|  |  |  |  |  | **LH7/AA5H1'** | 0.4475 | 0.0432 | 0.6000 | 0 |
|  |  |  |  |  | **LH4/BA5H4'** | 0.4285 | 0.0604 | 0.6000 | 0 |
|  |  |  |  |  | **LH3/BA5H1'** | 0.5289 | 0.0706 | 0.6000 | 0 |
|  |  |  |  |  | **LH7/AT4H2'(')** | 0.2478 | 0.0353 | 0.3250 | 0 |
|  |  |  |  |  | **LH7/AT4H1'** | 0.4455 | 0.0505 | 0.4500 | 0 |
